# A multimodal approach for establishing *ACTL6A* and *ERCC1* as chemoresistance genes in locally advanced head and neck cancer

**DOI:** 10.3389/fphar.2025.1541987

**Published:** 2025-05-29

**Authors:** Raushan Kumar Chaudhary, Prakash Patil, Vijith Vittal Shetty, Uday Venkat Mateti, Praveenkumar Shetty

**Affiliations:** ^1^ Department of Pharmacy Practice, NGSM Institute of Pharmaceutical Sciences (NGSMIPS), Nitte (Deemed to be University), Mangalore, Karnataka, India; ^2^ Central Research Laboratory, K.S. Hegde Medical Academy (KSHEMA), Nitte (Deemed to be University), Mangalore, Karnataka, India; ^3^ Department of Medical Oncology, K.S. Hegde Medical Academy (KSHEMA), Justice K.S. Hedge Charitable Hospital (JKSHCH), Nitte (Deemed to be University), Mangalore, Karnataka, India; ^4^ Department of Biochemistry, K.S. Hegde Medical Academy (KSHEMA), Nitte (Deemed to be University), Mangalore, Karnataka, India

**Keywords:** chemoradiotherapy, chemoresistance, cisplatin, DNA repair, drug repurposing, evidence

## Abstract

**Background:**

DNA is generally considered the ultimate target of cisplatin, so DNA repair has become the hallmark for cisplatin chemoresistance that is attributed to the poor overall survival (50%) among patients with head and neck cancer (HNC). As the efficacy of cisplatin is dose-dependent, we conducted the first study in an Asian population to characterize the DNA repair genes *ACTL6A* and *ERCC1* based on the dosing of cisplatin-based chemoradiotherapy (CRT).

**Methods:**

Locally advanced HNC (LAHNC) patients who were planning to undergo cisplatin-based CRT were enrolled in a prospective study to quantify the dose-dependent expressions of *ACTL6A* and *ERCC1* from peripheral blood mononuclear cells via quantitative polymerase chain reaction; these results were integrated with computational analysis and systematic review/meta-analysis to formulate evidence-based translation decisions. The Friedman test and Wilcoxon’s test were used to compare the expressions of the two genes before and after CRT, and Spearman’s rank correlation was used to find the correlation between *ACTL6A* and *ERCC1* expressions. All statistical analyses were performed using SPSS version 29.

**Results:**

A total of 77 LAHNC patients were enrolled in this study, of which 96.1% were men and 3.9% were women with a mean age of 52.88 ± 9.68 years. The median expressions of *ERCC1* were significantly increased (*p* < 0.001) after 50% (0.19) and 100% CRT (0.23) compared to the baseline value (0.14), whereas *ACTL6A* expression decreased from 4.77 to 3.87 after 50% CRT (*p* < 0.05) and increased to 5.43 after 100% CRT. From the computational analysis, *ACTL6A* and *ERCC1* were found to be overexpressed among HNC patients and observed to regulate 10 repair pathways. Overexpressions of *ERCC1* and *ACTL6A* were predicted to infiltrate the tumors with CD4^+^ cells, macrophages, dendritic cells, and B cells. The hazard ratios for overall survival were found to be 1.67 among the *ACTL6A* overexpressed and 1.82 among the *ERCC1* overexpressed HNC patients via computational analysis and meta-analysis, respectively. Furthermore, FDA-approved drugs like gemcitabine and panobinostat were found to be the best candidates for downregulating *ERCC1* and *ACTL6A* expressions based on binding affinities of −3.707 and −4.198 kcal/mol, respectively.

**Conclusion:**

The increased expressions of *ACTL6A* and *ERCC1* during/after cisplatin-based CRT are expected to mediate DNA repair leading to chemoresistance, which could result in poor overall survival in HNC patients. Thus, FDA-approved drugs like panobinostat and gemcitabine can be repurposed to target the chemoresistance genes *ACTL6A* and *ERCC1*, respectively.

## Graphical Abstract

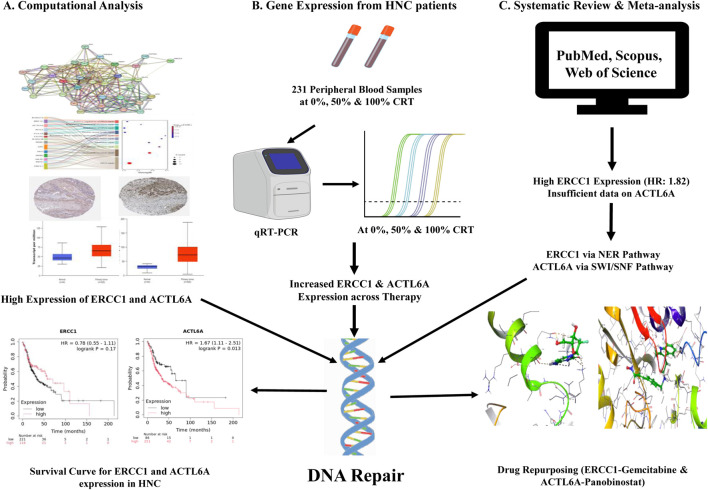

## Highlights

• Of the 77 LAHNC patients in the study cohort, men outnumbered women and had a mean age of 52.88 ± 9.68 years.
• *ACTL6A* expression increased after CRT (5.43) compared to the baseline value (4.77).
• *ERCC1* expression significantly increased with CRT, indicating high nucleotide excision repair capacity.
• *ERCC1*/*ACTL6A* overexpressions were linked to poor overall survival (hazard ratio: 1.82/1.67).
• Gemcitabine and panobinostat can downregulate *ERCC1* and *ACTL6A*, respectively.

## 1 Introduction

Head and neck cancer (HNC) refers to a group of heterogenous malignancies generally originating from the mucosal epithelial regions of the head and neck, such as the oral cavity, pharynx, larynx, oro/hypo/naso-pharynx, and salivary glands (Chaudhary et al., 2023). According to GLOBOCAN 2022, HNC has collectively secured the top spot among Indian patients in terms of incidence (17.53% or 247,924 new cases), 5-year prevalence (18.94% per 100,000 out of 613,841), and mortality (15.05% or 137,925) (Global Cancer Observatory, 2024: India Fact Sheet). Alcohol consumption, tobacco use (smoke/smokeless), poor oral hygiene, viral infections (human papilloma virus/Epstein–Barr virus), altered expressions of tumor suppressors, and oncogenes are the predominant etiopathophysiological factors associated with the development of HNC (Chaudhary et al., 2023). Localized/early-stage (stages I/II) HNCs are generally managed through surgery or radiation therapy, whereas locally advanced HNC (LAHNC) is generally managed using concurrent chemoradiotherapy (CCRT) with/without surgery (Chaudhary et al., 2023). Approximately 66.6% of the Indian population of HNC patients are diagnosed at the locally advanced stage, which makes CCRT with/without surgery as the popular choice of treatment among clinicians (Chaudhary et al., 2023; Mathur et al., 2020). Thus, chemotherapy serves as the cornerstone of the treatment strategy for managing HNC.

Cisplatin is the most widely preferred and broad-spectrum frontline dose-dependent antineoplastic drug in HNC that exerts its anticancer effects via the formation of interstrand and intrastrand cross-linking with nuclear/mitochondrial DNA at the N7 positions of adenine and guanine, thereby arresting the cell cycle at the G2 phase and causing apoptosis to interfere with DNA repair (Chaudhary et al., 2023; Ranasinghe et al., 2022). In addition, aqueous cisplatin is known to enhance the mitochondrial outer membrane permeabilization, which further induces the caspases and causes apoptosis of tumor cells through the release of protein cytochrome c into the cytoplasm (Kanno et al., 2021). Generally, once-weekly intravenous administration of cisplatin at 30–40 mg/m^2^ for 6–7 weeks has been proven to be the best alternative to 3-weekly intravenous administration of cisplatin at 100 mg/m^2^ as the former is associated with minimal toxicity (Chaudhary et al., 2023; NCCN Guidelines, 2024). Furthermore, cisplatin is often combined with other anticancer agents, such as paclitaxel, docetaxel, 5-fluorouracil (TPF regimen), hydroxyurea, etoposide, pembrolizumab, nivolumab, and cetuximab, to manage locally LAHNC and recurrent/metastatic HNC (R/MHNC) (Chaudhary et al., 2023; NCCN Guidelines, 2024). However, it is disheartening that almost 65% of LAHNC patients do not reap any benefits from such therapy, which is attributable to the recurrence, metastasis, and poor survival among LAHNC patients (Chaudhary et al., 2023; Mathur et al., 2020). Furthermore, approximately 70%–90% of R/MHNC patients do not respond to immunotherapy (Chaudhary et al., 2023). Collectively, these hurdles in the management of HNC have resulted in poor 5-year overall survival rates (50%) (Gormley et al., 2022). According to the Surveillance, Epidemiology, and End Results (SEER) registry, there has been a modest increase in the 5-year relative survival rate among HNC patients to approximately 65.25% between 2014 and 2020 (i.e., 5-year relative survival rates of oral cavity and pharynx cancer is 69% and larynx cancer is 61.5%) (SEER Cancer Stat Facts, 2024). The mortality rate of Indian patients accounts for approximately 71% of all HNC-related deaths in southeast Asia and 28% globally (Chauhan et al., 2018). The disease burden of HNC and its ineffective response to cisplatin have necessitated investigations into the causes behind the limited benefits of cisplatin therapy, which are achieved by exploring the possible biological markers involved in the molecular mechanisms of chemoresistance (Kanno et al., 2021).

Chemoresistance is a multifaceted condition that is often associated with increased DNA repair, deregulated influx/efflux pump, enzymatic inactivation of drugs, aberrant autophagy and apoptosis, regulation of EGFR/FAK/NF-kB pathways, cancer stem cells, and metabolic reprogramming (Ranasinghe et al., 2022; Kanno et al., 2021; Hu et al., 2023). As DNA is the ultimate target of cisplatin therapy, the pathways associated with repair of damaged DNA are crucially linked to cisplatin resistance. Nucleotide excision repair (NER) is a crucial DNA repair pathway that is responsible for clearing cisplatin-DNA adducts compared to other repair pathways, such as double-strand break repair, mismatch repair, and base excision repair (Kanno et al., 2021). NER is further subdivided into two important pathways, namely, the transcription-coupled repair (TCR-NER) and global genome repair (GGR-NER) pathways (KEGG Pathway, 2024: map03420). The current study explores the roles of the excision repair cross-complementation group1 (*ERCC1*) and actin-like protein 6A (*ACTL6A*) genes as attractive biological markers associated with DNA repair in HNC.

ERCC1 and ACTL6A are the core proteins of the NER pathway and switch/sucrose non-fermentable (SWI/SNF) complex, respectively (Kanno et al., 2021; Xiao et al., 2021). ERCC1 is a catalytically inactive protein that is capable of initiating DNA/protein–protein interaction (PPI) binds that can cause XPF activation and form the ERCC1-XPF1 heterodimer. Collectively, the ERCC1-XPF1 endonuclease protein complex is responsible for detection and repair of DNA damage. *ERCC1* is a high-capacity gene of the NER pathway that mediates cisplatin resistance in HNC patients (Prochnow et al., 2019). However, there exist controversies regarding its expression and clinical significance. Recently, the novel oncogene *ACTL6A* (a subunit of the SWI/SNF complex) has garnered attention for its DNA repair capacity (Xiao et al., 2021). Biologically, *ACTL6A* has been reported to be involved in chromatin remodeling and transcription regulation. *ACTL6A* encodes for the actin-related proteins comprising actin folds that are responsible for the binding and hydrolysis of adenosine triphosphate to remodel chromatin and promote gene expression by enhancing DNA accessibility (Xiao et al., 2021; Dang et al., 2020). Thus, *ACTL6A* mediates DNA repair via utilization of the SWI/SNF complex that might also promote such repair via NER (Dang et al., 2020). However, this mechanism remains unresolved.

**FIGURE 1:**
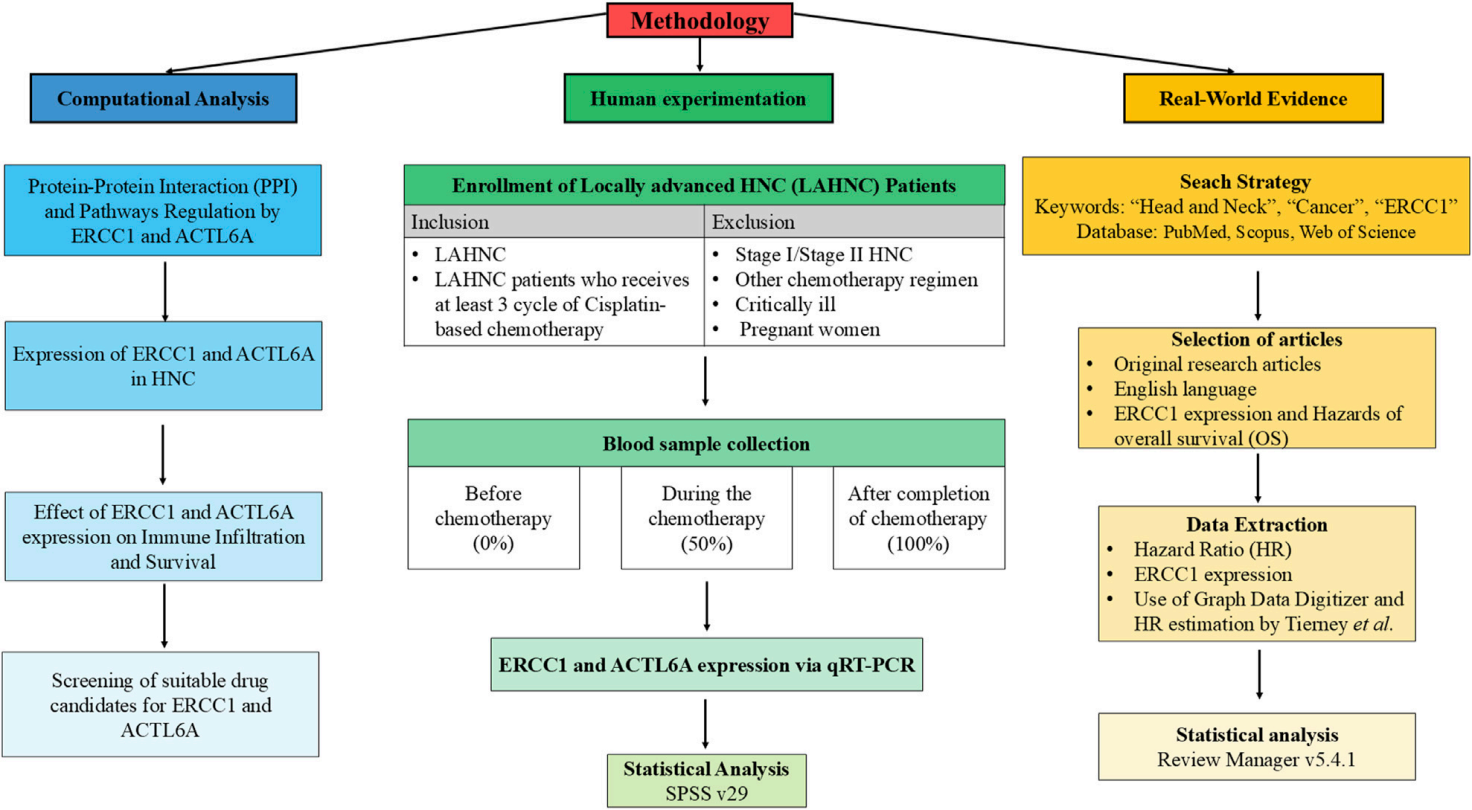
Detailed workflow showing integration of human experimentation with computational analysis and meta-analysis.

The formation of DNA–cisplatin adducts as well as the anticancer efficacy of cisplatin are attributed to the therapeutic dose administered (Ranasinghe et al., 2022). Thus, it is crucial to determine the biomarkers for cisplatin resistance in relation to the therapeutic dose. Furthermore, investigating the expressions of the chemoresistance genes from blood samples before and after therapy is less invasive, inexpensive, easy, less time-consuming, and ethically safe compared to using tissue samples that are difficult to obtain after therapy as this procedure may disturb the healing process or trigger recurrence, causing harm to the patient. Till date, there are no reported studies on detecting *ACTL6A* expression and very few studies on detecting *ERCC1* expression from blood samples. Furthermore, there are no available studies on characterizing the dose-dependent expressions of *ACTL6A* and *ERCC1* in HNC patients. Thus, to the best of our knowledge, this is the first study on Asian subjects to demonstrate the dose-dependent expressions of *ERCC1*/*ACTL6A* (zero cisplatin dose (zero cycle: 0 mg/m^2^), after administration of 50% dose (3-cycle cisplatin: 90 mg/m^2^), and after last cycle of cisplatin (4- or 5- or 6-cycle cisplatin: 120 or 150 or 180 mg/m^2^)) and their correlations at these three phases. Additionally, computational analysis and meta-analysis were performed to investigate regulation of the repair pathways through *ACTL6A* and *ERCC1* interactions with the platinum resistance genes to understand their expression patterns and impacts on overall survival to establish *ACTL6A* and *ERCC1* as the chemoresistance genes. The detailed workflow of the present study is depicted in Figure 1.

## 2 Materials and methods

### 2.1 Computational analysis to investigate *ACTL6A* and *ERCC1* in cisplatin resistance

#### 2.1.1 PPIs and pathway regulations of *ERCC1* and *ACTL6A*

In a previous study, we identified 21 genes that were regulated in the platinum drug resistance pathway (*ERCC1*, *MAPK1*, *MLH1*, *MDM2*, *PIK3CA*, *TP53*, *ERBB2*, *BAX*, *GSTM1*, *FAS*, *CASP8*, *FASLG*, *ABCC2*, *XIAP*, *BCL2*, *GSTP1*, *CDKN1A*, *TOP2A*, *CDKN2A*, *BRCA1*, and *BIRC2*) and five hub genes in cisplatin resistance (*CCND1*, *AXL*, *CDKN2A*, *TERT*, and *EZH2*), among which *ERCC1* was the only NER gene that was regulated for cisplatin resistance (Chaudhary et al., 2023). Furthermore, it has been reported that *ACTL6A* may contribute to DNA repair via the NER pathway, but its exact mechanism remains a mystery (Xiao et al., 2021). Thus, we investigated the interactions of *ACTL6A* with these 21 genes and the NER genes to clearly map the contributions of *ERCC1*/*ACTL6A* in DNA repair using STRING version 12.0 (https://string-db.org/). Furthermore, an unsupervised analysis was performed via K-means clustering to obtain similar protein clusters (https://string-db.org/) (Szklarczyk et al., 2023).

#### 2.1.2 mRNA and tissue expressions of *ERCC1* and *ACTL6A* in HNC

Overexpression of the DNA repair genes could contribute to the development of cisplatin resistance. Thus, *ERCC1* and *ACTL6A* were investigated for their mRNA- and tissue-level expressions using the UALCAN database (https://ualcan.path.uab.edu/analysis.html) (Chandrashekar et al., 2022, 2017) and Human Protein Atlas (https://www.proteinatlas.org/; The Human Protein Atlas, 2024), respectively. Furthermore, the *ERCC1* and *ACTL6A* genes were queried as target inputs in the muTarget platform to identify the top-5 genes undergoing somatic mutations with prevalence rates of at least 1% among the HNC patients while significantly overexpressing *ERCC1* and *ACTL6A* (https://www.mutarget.com/; Nagy and Győrffy, 2021). Here, muTarget is a platform that links gene expressions with the mutation statuses of the provided genes in solid cancers.

#### 2.1.3 Effects of *ERCC1* and *ACTL6A* expressions on immune infiltration and survival

The infiltration of cancer cells is often linked to compromised tumor responses to anticancer agents, leading to poor clinical outcomes. Thus, the impacts of *ERCC1* and *ACTL6A* expressions on immune cell infiltration in HNC were predicted using TIMER 2.0 (http://timer.cistrome.org/; Li et al., 2020); further, their effects on the overall survival of LAHNC patients (stages III and IV) were investigated using the Kaplan–Meier plotter (https://kmplot.com/analysis/; Győrffy, 2024). The survival analysis was independent of therapy as the Kaplan–Meier plotter database does not have the option to restrict analysis based on treatment time framework, i.e., pre- and post-therapy.

#### 2.1.4 Screening and binding of suitable drug candidates for *ERCC1* and *ACTL6A*

The DNA repair genes *ERCC1* and *ACTL6A* were screened for possible interactions with suitable FDA-approved or non-approved drug candidates using DGIbd (https://www.dgidb.org/; Cannon et al., 2024). Furthermore, the drug candidates were docked against their corresponding targets (*ERCC1* and *ACTL6A*) using Schrodinger version 2022-1 (https://www.schrodinger.com/) to investigate potential molecules other than platinum drugs that could downregulate *ERCC1* and *ACTL6A*. The protein structures of *ERCC1* (PDBID: 2A1I) and *ACTL6A* (PDBID: 9C4B) were obtained from protein data bank (https://www.rcsb.org/; RCSB PDB, 2024), and the structures of their corresponding drug candidates were obtained from PubChem database (https://pubchem.ncbi.nlm.nih.gov/; PubChem, 2024).

### 2.2 Human study for *ERCC1* and *ACTL6A* expressions among HNC patients

A prospective observational study was carried out at the Department of Oncology at a tertiary care hospital, where LAHNC patients above 18 years of age who were planning to undergo cisplatin-based CRT were enrolled after obtaining written informed consent. However, HNC patients with localized tumors (stage I/II) or those who were scheduled for other treatment modalities, critically ill patients, and pregnant women were excluded from the study. The study was initiated after obtaining approval from the Central Ethics Committee of the university (Ref. no. NU/CEC/2022/307 dated 21 September 2022 and revised on 31 January 2024 with Ref. no. NU/CEC/2024/526) and was also registered as a clinical trial in India (CTRI/2022/10/046142).

The sample size was calculated using the following formula for HNC prevalence of 30% (Dandekar et al., 2017; Prabhash et al., 2020) (P = 0.3) and marginal error of 9% (d = 0.09) at the 95% confidence interval (CI; Z_α/2_ = 1.96). Thus, the total number of HNC patients was calculated to be 99.59 (rounded to 100). However, only 66.6% of the people in this population 100 belong to the LAHNC group (Mathur et al., 2020). Hence, the minimum sample size required for the study was 67 (N). The final sample size to be enrolled was estimated to be 77 after adjusting the study population for a 15% dropout rate.

$$N={\left(\frac{Z_{\alpha /2}}{d}\right)}^{2}P\;\left(1-P\right)$$

#### 2.2.1 Blood sampling and clinical data collection

All LAHNC patients who were enrolled in the study had been scheduled to receive CCRT, i.e., six cycles of cisplatin at 30 mg/m^2^ weekly along with radiation of 60–70 Gy. After obtaining the informed consent and enrolling the participants, approximately 2 mL of peripheral blood sample was collected from each LAHNC patient in EDTA vacutainers and stored at −80°C. The patient blood was sampled at three different phases, i.e., zero cisplatin (before initiation of cisplatin-based CRT), after 50% of the planned cisplatin was administered (after third cycle of cisplatin therapy), and after completion or last cycle of cisplatin therapy. For patients who received only three cycles of cisplatin therapy, the third phase of blood sampling was conducted after completion of radiation therapy. Furthermore, we collected the demographic details and clinical characteristics of the patients.

#### 2.2.2 Primer selection, verification, and confirmation for *ERCC1* and *ACTL6A* genes

The primers for the quantitative real-time polymerase chain reaction (qRT-PCR) were obtained from PrimerBank (https://pga.mgh.harvard.edu/primerbank/; PrimerBank-MGH-PGA, 2023) and verified with the protein-coding regions of the cDNA sequences for selected transcripts of *ERCC1* and *ACTL6A* from the Ensembl database (https://www.ensembl.org/; Ensembl genome browser, 2023). The amplicon size of the primer selected for *ERCC1* was 175 base pairs (forward: TTT​GGC​GAC​GTA​ATT​CCC​GAC; reverse: CCT​GCT​GGG​GAT​CTT​TCA​CA) and that for *ACTL6A* was 83 base pairs (forward: GAC​AGC​ATT​TGC​TAA​TGG​TCG​T; reverse: CAT​CGT​GGA​CTG​GAA​TTG​CAG); further, the predesigned primer for β-actin (ACTB) had 249 base pairs (forward: CAT​GTA​CGT​TGC​TAT​CCA​GGC; reverse: CTC​CTT​AAT​GTC​ACG​CAC​GAT). The primers for *ERCC1* and *ACTL6A* along with the predesigned ACTB were confirmed experimentally via conventional PCR followed by DNA gel electrophoresis.

#### 2.2.3 *ERCC1* and *ACTL6A* expressions via PCR

##### 2.2.3.1 Total RNA extraction

The blood samples were centrifuged at 3,000 rpm to separate the plasma, followed by treatment of the blood cells with 1× RBC lysis solution. The mixture of blood cells and RBC lysis solution was left for 15–20 min to ensure RBC lysis and then centrifuged at 3,000 rpm to obtain Peripheral blood mononuclear cells (PBMC). The PBMC were used to extract the total RNA via the TRIzol reagent method using the RNAiso Plus kit (Takara, cat. no. 9109_v201904Da). The purity and concentration of the extracted RNA were confirmed via the nanodrop method.

##### 2.2.3.2 cDNA synthesis

cDNA was synthesized from the total RNA using the PrimeScript™ RT reagent kit (Takara, cat. no. RR037A_v202008Da) in a thermal cycler (Prima 96, HiMedia, India), i.e., reverse transcription was performed at 55°C for 60 min, followed by inactivation of reverse transcriptase at 87°C for 5 s and 4°C thereafter. The synthesized cDNA was stored at −20°C.

#### 2.2.4 qRT-PCR

The expressions of *ERCC1* and *ACTL6A* were obtained using TB Green Premix EX Taq (Tli RNase H Plus, Takara, cat. no. RR820A_ v201903Da) and quantified with the Applied Biosystems™ QuantStudio™ 6 RT-PCR System. The cDNA templates of the targets (*ERCC1* and *ACTL6A*) and reference (*ACTB*) were amplified using the QuantStudio™ system and SYBR Green PCR Master Mix fluorochrome dye. The qRT-PCR involved three stages: initial denaturation at 95°C for 3 min; PCR-based quantification at 95°C for 15 s followed by 60°C for 30 s and 72°C for 40 s (40 cycles); melting curve at 95°C for 15 s and 60°C for 1 min followed by 95°C for 15 s. The baseline and follow-up samples from a particular patient were processed together to avoid technical errors with the gene expressions. In addition, all samples were processed in duplicate along with RNAase-free water as the negative control.

## 3 Relative gene expressions and statistical analysis

The relative expressions of *ERCC1* and *ACTL6A* at baseline, after the third cycle of cisplatin therapy, and after the last cycle of cisplatin therapy were estimated by comparing the cycle threshold (Ct) value of a given sample for a particular gene of interest (GOI) (*ERCC1* and *ACTL6A*) with the Ct value of a given sample for the reference gene (*ACTB*). The relative expressions of the GOIs compared to the reference were calculated using the 2^−△CT^ formula [△CT = Ct (GOI) – Ct (ACTB)]. The patient data were analyzed using descriptive statistics (mean, standard deviation, frequency, percentage, and interquartile range) and were checked for normal distribution using the one-sample Kolmogorov–Smirnov test (*p* < 0.05). The *ERCC1* and *ACTL6A* expressions between the baseline and follow-ups were compared using the Friedman test. Furthermore, paired comparisons were performed via Wilcoxon’s test. Correlations between *ERCC1* and *ACTL6A* expressions were examined using Spearman’s rank test. All statistical analyses were performed using SPSS version 29 and figure was constructed using GraphPad Prism 8.0.2.

## 4 Real-world evidence for *ERCC1*/*ACTL6A* expressions and survival in HNC via meta-analysis

### 4.1 Research question and registration

Previous published meta-analyses by Xuelei et al. (2015) and Bišof et al. (2016) revealed that overexpression of *ERCC1* is the root cause of unfavorable overall survival outcomes (hazard ratio (HR): 2.14 and 1.95) among HNC patients, with the Asian population being the most affected victims (HR: 2.97 and 3.13, respectively). Thus, we conducted a further meta-analysis to update the predictive value of *ERCC1* on overall survival. This meta-analysis was registered prospectively with the International Prospective Register of Systematic Review (PROSPERO, 2024) under the title “Impact of ERCC1 expression on overall survival rate in head and neck cancer” with the registration ID CRD42024542859. However, data on the expression of *ACTL6A* and its impact on the survival of HNC patients are unavailable; thus, we could not conduct a meta-analysis for *ACTL6A*.

### 4.2 Search strategy

An electronic search was conducted for articles in the PubMed, Scopus, and Web of Science databases. The search strategy involved a combination of the following keywords: “Head and Neck,” “ERCC1,” “Cancer.” Further, “Head and Neck,” “ACTL6A,” and “Cancer” were used as the keywords to retrieve articles related to the *ACTL6A* gene in HNC (see also the Supplementary File). In addition, the reference citations in these articles were manually checked for additional studies. Rayyan Software was used to import and manage the articles.

### 4.3 Eligibility criteria and selection process

The studies included in this meta-analysis/review were original research articles published in English language that evaluated the relationships between overall survival rate and expression of *ERCC1* or *ACTL6A* in HNC. After removing duplicate articles, the title and abstract were screened for eligibility by two independent authors. Any disagreements between these authors were resolved by a third author after a consensus discussion. Later, the selected studies were assessed for eligibility by two different authors based on the full text, and any disagreements were resolved by a third author. Articles without full text and ineligible articles were excluded from the study.

### 4.4 Data extraction

Data were extracted from eligible articles using the predesigned proforma containing the following information: author details, country, year of publication, sample size, gender, age, study design, disease details, TNCM/clinical staging, molecular technique used, *ERCC1* expression, and outcomes of the study. The data were extracted by two independent authors, and any disagreements were resolved by a third author. If the survival data were represented using the Kaplan–Meier curve, and then the relevant information was interpreted from the graph using Graph Data Digitizer 2.4. If the HR was not reported by the authors, then it was estimated using the method proposed by Tierney et al. (2007).

### 4.5 Statistical analysis

Review Manager software v5.4.1 (Cochrane Collaboration, Copenhagen, Denmark) was used to generate the forest plots, and the inverse variance method was used for pooled estimates. The outcome variables of all the included studies were represented in terms of the HR and 95% CI. The analyses were performed using RevMan calculator by incorporating the log(HR) with standard error (SE), and the results were presented as HR with 95% CI. All results were presented graphically and numerically in the forest plot along with the weights imparted by the individual studies. The Higgins I^2^ statistic and visual inspection were used to assess heterogenicity, and the percentage with *p*-value was used to represent interstudy variability. Both random and fixed effects were used; the fixed-effects model was used when the percentage of heterogenicity was I^2^ ≤ 40%, whereas the random effects model was used when I^2^ > 40%. Furthermore, publication bias was assessed using funnel plots and Egger’s regression test.

## 5 Results

### 5.1 Computational analysis to investigate cisplatin resistance in HNC via *ERCC1*/*ACTL6A*

#### 5.1.1 PPIs associated with *ERCC1*/*ACTL6A* and enrichment analysis

**FIGURE 2:**
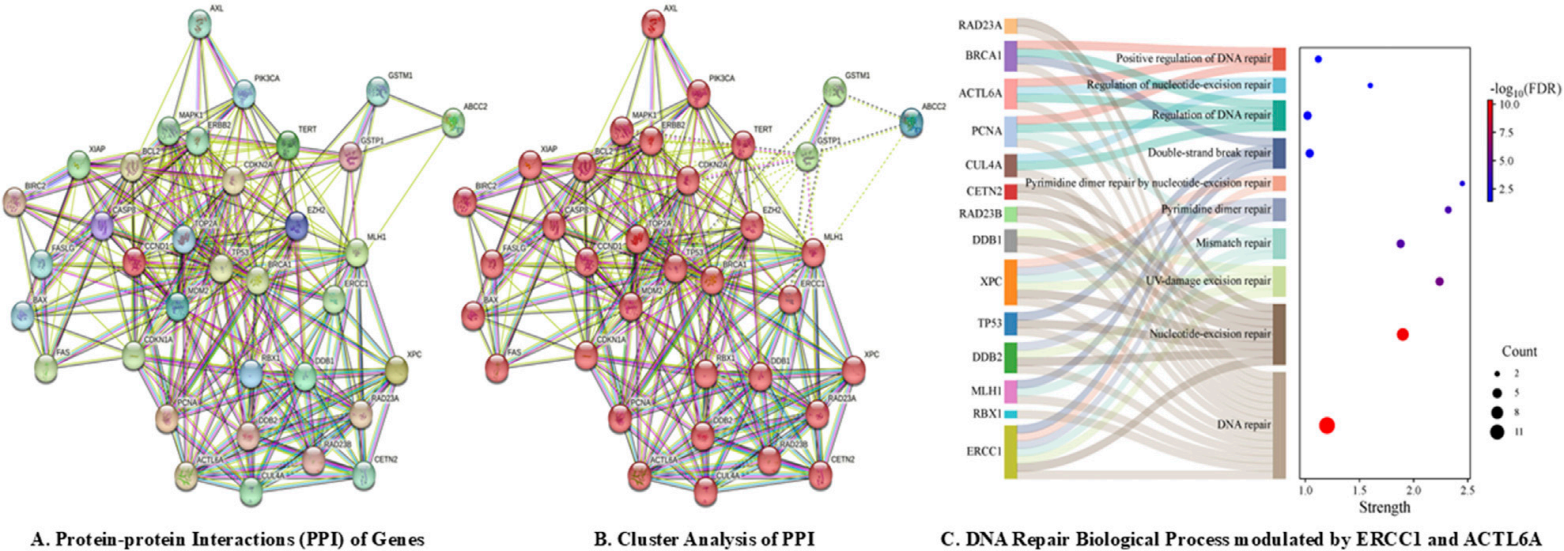
DNA repair pathways modulated by *ACTL6A* and *ERCC1* via computational analysis: **(A)** protein–protein interactions; **(B)** cluster analysis; **(C)** biological process modulations by *ERCC1* and *ACTL6A*.

A total of 35 genes were investigated for PPIs, including 21 genes for platinum drug resistance, *ACTL6A*, and eight genes for GGR-NER, with medium confidence (0.400); this revealed interactions between 30 nodes, resulting in a total of 260 edges at an average node degree of 14.9 and average local clustering coefficient of 0.737 (Figure 2A). The PPIs significantly enriched (<1.0e^−16^) 177 biological process (BP), 17 cellular component (CC), and 15 molecular function (MF) terms of gene ontology along with four KEGG pathways that were modulated by *ERCC1* and/or *ACTL6A*. The *ACTL6A* gene was found to significantly modulate BPs such as DNA repair, regulation of DNA repair, positive regulation of DNA repair, and regulation of NER, whereas the *ERCC1* gene was found to modulate BPs like DNA repair, NER, UV damage excision repair, mismatch repair, pyrimidine dimer repair, pyrimidine dimer repair by NER, and double-strand break repair (Figure 2C). All these repair BPs were modulated via the PPIs of 14 identical genes belonging to the same cluster (red color), namely, *ACTL6A*, *ERCC1*, *PCNA*, *MLH1*, *BRCA1*, *TP53*, *XPC*, *CETN2*, *CUL4A*, *DDB1*, *DDB2*, *RAD23A*, *RAD23B*, and *RBX1* (Figure 2B). Additionally, the DNA repair and NER complexes were the CCs modulated by *ERCC1*. This shows that both *ERCC1* and *ACTL6A* are involved in DNA repair processes, particularly via the NER pathway.

#### 5.1.2 mRNA and tissue expressions of *ERCC1* and *ACTL6A* in HNC

**FIGURE 3:**
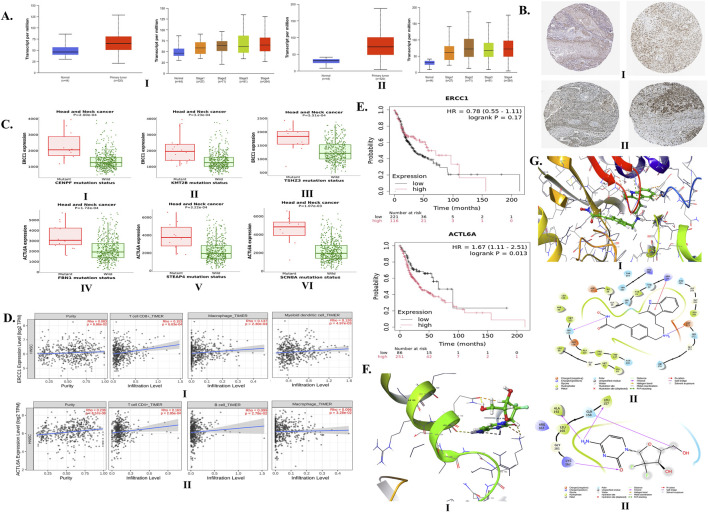
Expressions of *ERCC1* and *ACTL6A* in head and neck cancer (HNC) and their impacts on tumor infiltration and overall survival via computational analysis. **(A)** mRNA expressions of **(I)** *ERCC1* and **(II)** *ACTL6A* in tumor vs. normal samples based on stage of HNC. **(B)** Expressions of **(I)** *ERCC1* and **(II)** *ACTL6A* in tumor tissues via immunohistochemistry. **(C)** Gene mutations upregulating **(I–III)** *ERCC1* and **(IV–VI)** *ACTL6A* expressions. **(D)** Infiltration of **(I)** CD8^+^ cells, macrophages, and myeloid dendritic cells by *ERCC1* expression and **(II)** CD4^+^ cells, B cells, and macrophages by *ACTL6A* expression. **(E)** Impacts of *ERCC1* and *ACTL6A* expressions on overall survival. **(F) (I)** 3D and **(II)** 2D interactions of gemcitabine with *ERCC1*. **(G) (I)** 3D and **(II)** 2D interactions of panobinostat with *ACTL6A*.

The median transcripts per million of *ERCC1* and *ACTL6A* were found to be significantly higher in primary tumors (64.801 and 71.98, respectively) than normal samples (46.256 and 30.826, respectively) (Figure 3A). Both *ERCC1* and *ACTL6A* were found to be overexpressed in advanced stages except stage III HNC patients (stage IV > stage II > stage III > stage I) (Figure 3B), particularly among African-American and Caucasian people compared to Asian patients. Furthermore, *ERCC1* was found to be overexpressed greatly among persons aged 61–80 years, followed by those in the age groups of 41–60 years, 81–100 years, and 21–40 years; however, *ACTL6A* was found to be overexpressed greatly among persons aged 41–60 years, followed by those in the age groups of 61–80 years, 21–40 years, and 81–100 years. Additionally, *ERCC1* is inconsistently expressed with advancing tumor grade (grade 3 > grade 2 > grade 4 > grade 1), whereas *ACTL6A* shows significant overexpression with the grade of tumor progression (grade 4 > grade 3 > grade 2 > grade 1) (see Supplementary Table S1 and Supplementary Figure S1). Thus, we can predict that both *ERCC1* and *ACTL6A* are upregulated in HNC patients, particularly in individuals with advanced stages and tumor grades and mostly in persons aged 40–80 years.

Furthermore, *ERCC1* and *ACTL6A* were found to show moderate and moderate-to-strong expressions, respectively. The immunohistochemistry (IHC) report of HNC tissue enclosed in HPA shows moderate expression of *ERCC1* at nuclear-level staining using antibodies, such as HPA029773, CAB004390, CAB072859, and CAB072860 (Figure 3B.I and Supplementary Figure S2). Furthermore, *ACTL6A* shows moderate-to-strong expression at the nuclear- and cytoplasmic/membranous-nuclear-level staining using the CAB012188 antibody (Figure 3B.II, Supplementary Figure S2). These findings confirm the expressions of *ERCC1* and *ACTL6A* in various types of HNC tumor tissues. Moreover, there are certain mutations observed in HNC patients that can alter the expressions of *ERCC1* and *ACTL6A*. The top-5 genes undergoing somatic mutations with at least 1% prevalence rates contribute to the overexpressions of *ERCC1* (namely, *CASK*, *CENPF*, *KMT2B*, *TSHZ3*, and *DVL1*) and *ACTL6A* (namely, *FBN1*, *STEAP4*, *SCN8A*, *OR8H2*, and *CASZ1*) (Figure 3C, Supplementary Tables S2, S3, Supplementary Figures S3, S4).

#### 5.1.3 Impacts of *ERCC1* and *ACTL6A* expressions on tumor cell infiltration and survival in HNC

The overexpressions of *ERCC1* and *ACTL6A* were found to significantly enhance the infiltration of CD8^+^ T-cells, macrophages, dendritic cells, CD4^+^ T-cells, and B-cells into HNC tumor cells (Figure 3D, Supplementary Figure S5). Overexpression of *ERCC1* was found to be linked with 22% less risk of death compared to reduced expression in HNC patients (HR: 0.78, *p* = 0.17), whereas overexpression of *ACTL6A* was found to be linked with 67% more risk of death among HNC patients (HR: 1.67, *p* = 0.013) (Figure 3E). Thus, computational analysis reveals that *ACTL6A* is a significant gene responsible for the poor survival of HNC patients.

#### 5.1.4 Potential drug candidates and their binding affinities with *ERCC1* and *ACTL6A*

A total of 12 drug candidates, i.e., eight FDA-approved drugs (cyclosporine, carboplatin, cisplatin, 5-fluorouracil, doxorubicin hydrochloride, gemcitabine, paclitaxel, and thalidomide) and four drugs not approved by the FDA (staurosporine, herbimycin A, platinum, and platinum compound), were found to interact with the ERCC1 protein. Similarly, a total of four drug candidates (two FDA-approved drugs: panobinostat and cisplatin; two unapproved drugs: sphingosine-1-phosphate and sphingosylphosphorylcholine) were found to interact with the ACTL6A protein (Supplementary Table S4). In the case of cisplatin resistance, the predicted FDA-approved drug candidates other than platinum may be repurposed to downregulate both *ERCC1* and *ACTL6A* genes. Thus, gemcitabine (interaction score: 0.047) and paclitaxel (interaction score: 0.040) were found to have high interactions with *ERCC1*, whereas panobinostat (interaction score: 0.398) was found to interact with *ACTL6A*. From the molecular docking studies, we found that gemcitabine and panobinostat possessed higher binding affinities toward *ERCC1* and *ACTL6A* with binding energies of −3.707 kcal/mol (Figure 3F) and −4.198 kcal/mol (Figure 3G), respectively.

### 5.2 Human study for *ERCC1* and *ACTL6A* expressions among HNC patients

#### 5.2.1 Demographic details and clinical characteristics of the HNC patients

A total of 77 LAHNC patients were enrolled in the study, of which 96.1% patients were men and 3.9% were women with a mean age of 52.88 ± 9.68 years. The majority of patients were in the age group of 41–60 years (61.04%), followed by 61–80 years (29.87%) and 21–40 years (9.09%). Nearly half of the enrolled patients (49.35%) were from the upper part of the lower socioeconomic class and had abnormal body mass index (BMI) values, i.e., they were underweight (42.86%) or overweight (6.49%). Most of the LAHNC patients (89.61%) had a history of social habits, such as drinking alcohol (58.44%), smoking tobacco (46.75%), or chewing tobacco (38.96%) or betel leaf (33.77%) or areca nut (33.77%). Approximately 16.88% of the patients reported both alcohol consumption and smoking. Furthermore, we observed that approximately 24.68% of the HNC patients had comorbidities, where hypertension (15.58%) and diabetes (11.69%) were the most prevalent types followed by cerebrovascular accidents (5.19%), respiratory diseases (2.60%), and ischemic heart disease (1.30%). We found that approximately 29.87% of patients had a history of cancer in their family. Surprisingly, HNC was the most commonly reported type of cancer (15.58%) in the family histories, which was attributed to prevailing social habits in their families (Table 1).

**TABLE 1:** Demographic details and clinical characteristics of the HNC patients in this study.

Parameters	Frequency	Percentage (%)
Gender		
Male	74	96.1
Female	3	3.90
Age Groups (52.88 ± 9.68 years)		
21–40 years	7	9.09
41–60 years	47	61.04
61–80 years	23	29.87
BMI Categories (19.39 ± 3.72)		
Overweight	5	6.49
Normal BMI	39	50.65
Underweight	33	42.86
Kuppuswamy Socioeconomic Class		
Lower Middle	30	38.96
Upper Lower	38	49.35
Upper Middle	9	11.69
Social Habits		
No habits	7	9.09
Alcohol consumption	45	58.44
Tobacco smoking	36	46.75
Tobacco chewing	30	38.96
Betel leaf or paan chewing	26	33.77
Areca nut or gutka chewing	26	33.77
Sharp Teeth Associated Injury		
Tongue bite	7	9.09
Cheek bite	5	6.49
Comorbidities		
No comorbidities	58	75.32
Hypertension	12	15.58
Diabetes mellitus	9	11.69
Cerebrovascular accident	4	5.19
Respiratory diseases: Asthma, COPD, and Old TB	2	2.60
Ischemic heart disease	1	1.30
Family History of Cancer		
No familial history of cancer	54	70.13
Patients with familial history of cancer	23	29.87
*Breast*	1	1.30
*Breast and Brain*	1	1.30
*Hematological*	3	3.90
*HNC*	13	16.88
*HNC and Breast*	1	1.30
*Thyroid*	1	1.30
*Uterus*	2	2.60
*Brain*	1	1.30
Histopathology		
Well differentiated (Grade 1)	27	35.06
Moderately differentiated (Grade 2)	43	55.84
Poorly differentiated (Grade 3)	7	9.09
8th Edition of the American Joint Committee on Cancer (AJCC) staging		
Stage III	16	20.78
Stage IV	61	79.22
Stage IVA	47	61.04
Stage IVB	14	18.18
Types of HNC		
**Oral Cavity Cancer**	40	51.95
*Buccal mucosa cancer*	13	16.88
*Tongue cancer*	18	23.38
*Floor of the mouth*	3	3.90
*Gingivobuccal sulcus*	1	1.30
*Hard palate*	1	1.30
*Retromolar trigone*	4	5.19
**Laryngeal cancer**	13	16.88
*Supraglottic cancer*	5	6.49
*Aryepiglottic cancer*	2	2.60
*Epiglottic cancer*	1	1.30
*Vocal cord cancer*	5	6.49
**Hypopharynx cancer**	11	14.29
*Cricopharynx*	2	2.60
*Pyriform fossa*	9	11.69
**Oropharynx cancer**	8	10.39
*Base of tongue cancer*	5	6.49
*Soft palate*	3	3.90
**Cancer of unknown primary cause**	3	3.90
*Lymph node*	2	2.60
*Brachial cleft cyst*	1	1.30
**Nasopharynx cancer** (Nasal cavity)	2	2.60
Treatment Modalities		
Total concurrent CRT (CCRT)	55	71.43
Concurrent CRT (CCRT)	46	59.74
CCRT with adjuvant chemotherapy	9	11.69
Surgery with adjuvant CCRT	22	28.57
Number of Cycles of Cisplatin Chemotherapy		
3	8	10.39
4	6	7.79
5	44	57.14
6	19	24.68
Radiation Dose		
60 Gy	17	22.08
66 Gy	43	55.84
70 Gy	17	22.08
Radiation Fraction		
30	23	29.87
33	37	48.05
35	17	22.08

Clinically, the majority of the enrolled LAHNC patients belonged to grade 2 (55.84%) followed by grades 1 and 3 and were diagnosed at stage IV (79.22%) followed by stage III (20.78%). Approximately half of the HNC patients were diagnosed with carcinoma of the oral cavity (51.95%), followed by laryngeal cancer (16.88%), hypopharyngeal cancer (14.29%), oropharyngeal cancer (10.39%), cancer of unknown primary cause (3.90%), and nasopharyngeal cancer (2.60%). CCRT was the most popular choice of treatment (55.84%), followed by surgery with adjuvant CRT (32.47%) and CCRT with adjuvant chemotherapy (11.69%). All patients in the study cohort were scheduled to undergo six cycles of cisplatin therapy. However, approximately half of the patients received five cycles of cisplatin (57.14%), followed by six cycles (24.68%), three cycles (10.39%), and four cycles (7.79%). The dosage for radiation therapy ranged from 60 to 70 Gy and was administered in 30–35 fractions. The demographics and clinical characteristics of the LAHNC patients are depicted in Table 1.

#### 5.2.2 *ERCC1* and *ACTL6A* expressions from peripheral blood samples via qPCR

Considering the expression of the reference gene as 1 (with >1 being high expression and <1 being low expression), *ERCC1* was highly expressed among 14.29% patients out of the total of 77 HNC patients while 85.71% of patients showed low expressions compared to the baseline. Furthermore, 9.09% and 20.78% of patients were observed to have higher expressions of *ERCC1* after 50% CCRT and 100% CCRT, respectively. Similarly, *ACTL6A* was highly expressed in 88.31% of the patients while 11.69% of the patients had low expressions compared to the baseline. After administration of 50% and 100% CCRT dosing, *ACTL6A* expressions were found to be highly expressed among 75.32% and 84.42% of the patients, respectively (Supplementary Table S5). This shows that cisplatin-based CCRT initially decreases the expressions of *ERCC1* (14.29%–9.09%) and *ACTL6A* (88.31%–75.32%) among HNC patients via initial response to therapy, whereas the expressions of *ERCC1* (9.09%–20.78%) and *ACTL6A* (75.32%–84. 42%) increase later to confer possible resistance to cisplatin therapy.

**FIGURE 4:**
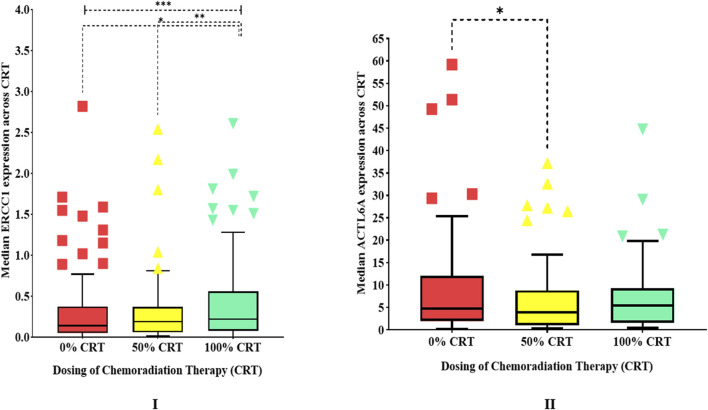
Human experimentation results showing box plots of **(I)** *ERCC1* expressions (outliers with median expressions >4 have been removed) and **(II)** *ACTL6A* expressions over the duration of chemoradiotherapy.

Comparative analyses on the impacts of cisplatin-based CCRT on the gene expressions showed that the overall median expression of *ERCC1* significantly increased (*p* < 0.001) by 1.64-fold compared to the baseline (from 0.14 to 0.19 and 0.23), signifying that *ERCC1* could potentially be involved in DNA repair (Table 2 and Figure 4.I). Similarly, the median expression of *ACTL6A* significantly decreased by 0.81-fold (from 4.77 to 3.87) after the initial three cycles of CCRT but later increased by 1.14-fold (from 3.87 to 5.43), showing the ability of *ACTL6A* to bounce back and mediate DNA repair (Table 2 and Figure 4.II). Furthermore, the subgroup analysis of variables showed that patients with advanced ages (40–80 years), advanced stages (stage IV), highly differentiated tumors (grades 1 and 2), low BMIs (underweight/normal), social habits (tobacco smoking, alcohol consumption, betel leaf chewing), oral cavity cancers, and hypopharyngeal cancer who received CCRT alone or five cycles of cisplatin are at high risk of developing ERCC1-mediated cisplatin resistance as ERCC1 was found to be significantly increased in these patients. In contrast, patients with no history of tobacco use or betel leaf chewing also showed significant increases in *ERCC1* expressions. Interestingly, we observed that *ACTL6A* expressions were significantly lower in patients with no history of tobacco smoking, alcohol consumption, or tobacco/betel leaf/areca nut/gutka chewing (Table 2). This indicates that patients with a history of social habits may be at a greater risk of developing chemoresistance to CCRT than patients without such history. Additionally, correlation analysis did not indicate any correlation in the baseline expressions of *ERCC1* and *ACTL6A* (ρ = 0.201, *p* = 0.08). However, the expressions of these genes were significantly (ρ = 0.331, *p* = 0.003) and marginally (ρ = 0.215, *p* = 0.060) correlated after receiving 50% and 100% cisplatin-based CCRT, indicating that *ACTL6A* could indirectly influence DNA repair via the NER pathways.

**TABLE 2:** Comparison of *ERCC1* and *ACTL6A* expressions across chemoradiotherapy.

Parameters	Median expressions of genes						
Genes	0% CRT	50% CRT	100% CRT	*p*-value (0% vs. 50%)	*p*-value (50% vs. 100%)	*p*-value (0% vs. 100%)	Overall *p*-value
*ERCC1*	0.14 (0.05, 0.41)	0.19 (0.06, 0.44)	0.23 (0.08, 0.68)	0.301	0.001**	0.011*	p < 0.001***
*ACTL6A*	4.77 (1.92, 12.065)	3.87 (1.00, 8.81)	5.43 (1.535, 9.26)	0.028*	0.459	0.362	0.729

Sociodemographic/Clinical	Median Expression of *ERCC1* and *ACTL6A*						
Age Groups							
21–40 years *ERCC1*	0.12 (0.04, 0.36)	0.19 (0.08, 0.23)	0.12 (0.07, 0.32)	0.397	0.672	0.499	0.651
21–40 years *ACTL6A*	11.89 (1.12, 21.88)	6.39 (0.48, 9.82)	4.71 (1.42, 14.57)	0.128	0.612	0.499	0.565
41–60 years *ERCC1*	0.22 (0.05, 0.6)	0.17 (0.06, 0.48)	0.34 (0.13, 0.1.05)	0.782	0.005*	0.077	0.005*
41–60 years *ACTL6A*	4.49 (2.21, 11.73)	5.93 (2.61, 9.07)	6.78 (2.53, 9.95)	0.800	0.582	0.691	0.587
61–80 years *ERCC1*	0.01 (0.05, 0.32)	0.19 (0.04, 0.35)	0.21 (0.08, 0.37)	0.217	0.079	0.068	0.009*
61–80 years *ACTL6A*	4.76 (1.42, 11.98)	1.14 (0.77, 6.05)	2.06 (0.85, 7.16)	0.007	0.783	0.066	0.199
BMI							
Underweight *ERCC1*	0.11 (0.05, 0.385)	0.12 (0.06, 0.25)	0.2 (0.075, 0.56)	0.543	0.031*	0.136	0.029*
Underweight *ACTL6A*	5.19 (1.23, 11.55)	3.15 (0.81, 7.15)	4.37 (1.54, 8.90)	0.183	0.432	0.357	0.754
Normal *ERCC1*	0.15 (0.05, 0.6)	0.20 (0.06, 0.44)	0.26 (0.12, 0.88)	0.619	0.033*	0.121	0.021*
Normal *ACTL6A*	4.05 (2.14, 11.89)	4.25 (1.05, 8.84)	5.84 (1.17, 9.95)	0.264	0.596	0.967	0.975
Overweight *ERCC1*	0.27 (0.04, 0.345)	0.19 (0.085, 1.32)	0.51 (0.21, 2.975)	0.225	0.08	0.068	0.076
Overweight *ACTL6A*	21.59 (7.02, 25.62)	9.04 (5.89, 12.75)	6.78 (4.89, 12.89)	0.080	0.686	0.225	0.549
Social Habits							
No habits *ERCC1*	0.25 (0.09, 0.40)	0.10 (0.04, 0.14)	0.10 (0.05, 0.31)	0.236	0.610	0.917	0.772
Yes habits *ERCC1*	0.13 (0.05, 0.41)	0.19 (0.06, 0.45)	0.26 (0.097, 0.89)	0.153	0.001**	0.007*	p < 0.001***
No habits *ACTL6A*	11.73 (2.78, 21.59)	3.10 (1.14, 9.04)	8.37 (4.46, 10.63)	0.063	0.176	1.00	0.565
Yes habits *ACTL6A*	4.62 (1.67, 11.95)	3.88 (.937, 8.79)	5.41 (1.38, 9.14)	0.089	0.652	0.366	0.876
No smoking *ERCC1*	0.26 (0.085, 0.96)	0.17 (0.06, 0.44)	0.21 (0.095, 1.09)	0.559	0.003*	0.340	0.032
Smoking *ERCC1*	0.095 (0.05, 0.25)	0.195 (0.07, 0.44)	0.265 (0.08, 0.62)	0.016*	0.087	0.003*	0.001**
No Smoking *ACTL6A*	6.36 (2.66, 14.13)	4.87 (0.95, 9.06)	7.04 (3.19, 10.60)	0.013*	0.390	0.496	0.552
Smoking *ACTL6A*	3.68 (1.33, 9.71	3.315 (1.16, 7.62)	4.695 (1.09, 7.93)	0.530	0.888	0.599	1.000
No Alcohol consumption *ERCC1*	0.24 (0.09, 0.64)	0.11 (0.04, 0.38)	0.21 (0.07, 0.67)	0.206	0.014*	0.742	0.103
Alcohol consumption ERCC1	0.11 (0.04, 0.30)	0.19 (0.09, 0.30)	0.32 (0.125, 0.78)	0.011*	0.024*	0.002*	p < 0.001***
No alcohol consumption *ACTL6A*	4.27 (1.49, 11.52)	2.75 (0.84, 7.30)	5.26 (1.17, 9.82)	0.166	0.176	0.601	0.680
Alcohol consumption *ACTL6A*	6.27 (2.44, 13.72)	5.93 (1.48, 9.27)	5.59 (1.95, 8.17)	0.079	0.906	0.09	0.766
No tobacco chewing *ERCC1*	0.11 (0.04, 0.26)	0.19 (0.06, 0.29)	0.25 (0.08, 0.61)	0.137	0.002*	0.006*	p < 0.001***
Tobacco chewing *ERCC1*	0.28 (0.067, 0.89)	0.19 (0.06, 0.78)	0.22 (0.13, 1.17)	0.982	0.094	0.447	0.126
No tobacco chewing *ACTL6A*	4.05 (1.57, 11.98)	3.10 (1.05, 7.49)	5.15 (1.17, 8.78)	0.030*	0.174	0.608	0.722
Tobacco chewing *ACTL6A*	6.01 (2.54, 12.54)	6.74 (0.82, 11.45)	6.97 (1.89, 11.38)	0.441	0.586	0.417	0.905
No betel leaf chewing *ERCC1*	0.14 (0.05, 0.41)	0.19 (0.06, 0.29)	0.22 (0.08, 0.51)	0.927	0.007*	0.176	0.009*
Betel leaf chewing *ERCC1*	0.13 (0.037, .0397)	0.205 (0.06, 0.72)	0.44 (0.115, 1.32)	0.092	0.029*	0.014*	0.002*
No Betel leaf chewing *ACTL6A*	4.77 (1.41, 11.73)	3.77 (1.14, 8.42)	4.46 (1.17, 8.26)	0.033*	0.940	0.275	0.662
Betel leaf chewing *ACTL6A*	4.8 (3.21, 13.54)	6.1 (0.86, 9.04)	6.91 (3.53, 14.87)	0.439	0.382	0.929	0.832
No areca nut or gutka chewing *ERCC1*	0.14 (0.05, 0.40)	0.17 (0.06, 0.44)	0.31 (0.14, 0.88)	0.234	0.010*	0.008*	p < 0.001***
Areca nut or gutka chewing *ERCC1*	0.12 (0.047, 0.537)	0.20 (0.045, 0.44)	0.195 (0.07, 0.61)	0.957	0.020*	0.493	0.137
No areca nut or gutka chewing *ACTL6A*	7.37 (2.68, 13.73)	5.50 (1.14, 9.47)	5.43 (1.29, 9.02)	0.034*	0.593	0.058	0.494
Areca nut or gutka chewing *ACTL6A*	3.26 (1.22, 6.61)	2.99 (0.85, 7.49)	5.49 (1.59, 11.56)	0.657	0.035*	0.209	0.173
Comorbidity							
No comorbidity *ERCC1*	0.13 (0.05, 0.465)	0.19 (0.077, 0.48)	0.225 (0.09, 0.97)	0.227	0.006*	0.038*	0.003*
No comorbidity *ACTL6A*	3.83 (1.42, 12.49)	3.82 (1.06, 8.93)	5.72 (1.59, 9.61)	0.277	0.619	0.642	0.852
Presence of comorbidity *ERCC1*	0.15 (0.03, 0.32)	0.12 (0.03, 0.23)	0.31 (0.05, 0.51)	0.948	0.036*	0.121	0.011*
Presence of comorbidity *ACTL6A*	5.19 (3.76, 11.73)	3.89 (0.96, 7.50)	5.33 (1.29, 8.27)	0.027*	0.601	0.398	0.229
Stages							
Stage III *ERCC1*	0.245 (0.08, 1.27)	0.21 (0.045, 0.43)	0.19 (0.08, 0.55)	0.535	0.211	0.623	0.867
Stage III *ACTL6A*	2.71 (0.90, 11.91)	3.83 (1.33, 8.54)	5.62 (3.47, 14.31)	0.756	0.148	0.469	0.269
Stage IV *ERCC1*	0.12 (0.05, 0.395)	0.19 (0.06, 0.435)	0.27 (0.095, 0.78)	0.153	0.002*	0.001**	p < 0.001***
Stage IV *ACTL6A*	5.11 (2.44, 12.82)	3.87 (.915, 8.91)	5.43 (1.35, 8.90)	0.027*	0.947	0.156	0.452
Histopathology							
Poorly differentiated *ERCC1*	0.29 (0.09, 1.15)	0.26 (0.14, 0.84)	0.25 (0.10, 1.57)	0.672	0.446	0.866	0.651
Poorly differentiated *ACTL6A*	2.68 (1.41, 7.73)	4.25 (2.89, 8.89)	3.85 (1.15, 8.78)	0.612	0.866	0.398	0.368
Moderately differentiated *ERCC1*	0.12 (0.05, 0.36)	0.15 (0.06, 0.40)	0.21 (0.09, 0.95)	0.974	0.001**	0.053	0.001**
Moderately differentiated *ACTL6A*	6.27 (2.93, 13.49)	3.15 (0.94, 8.78)	5.89 (1.93, 9.02)	0.001**	0.358	0.098	0.108
Well differentiated *ERCC1*	0.14 (0.04, 0.42)	0.20 (0.06, 0.43)	0.27 (0.08, 0.68)	0.082	0.237	0.038*	0.030*
Well differentiated *ACTL6A*	4.05 (1.25, 13.7)	6.05 (.75, 8.84)	5.18 (1.17, 13.90)	0.829	1.00	0.848	0.772
Type of HNC							
Oral cavity cancer *ERCC1*	0.13 (0.05, 0.69)	0.185 (0.06, 0.44)	0.29 (0.12, 1.03)	0.632	0.017*	0.050*	0.007*
Oral cavity cancer *ACTL6A*	7.57 (2.48, 13.72)	6.43 (1.97, 11.03)	7.33 (1.45, 14.40)	0.162	0.707	0.364	0.928
Laryngeal cancer *ERCC1*	0.22 (0.04, 0.36)	0.15 (0.05, 0.34)	0.22 (0.05, 0.43)	0.506	0.208	0.649	0.146
Laryngeal cancer *ERCC1*	4.77 (1.32, 9.55)	2.84 (0.94, 7.59)	5.33 (2.39, 6.94)	0.249	0.753	0.701	0.584
Hypopharyngeal cancer *ERCC1*	0.09 (0.04, 0.36)	0.10 (0.06, 0.70)	0.15 (0.06, 1.14)	0.046*	0.333	0.139	0.027*
Hypopharyngeal cancer *ERCC1*	3.38 (0.66, 7.36)	5.50 (0.80, 7.50)	4.46 (0.80, 8.78)	0.859	0.721	0.929	0.368
Oropharyngeal cancer *ERCC1*	0.085 (0.027, 0.24)	0.24 (0.19, 0.28)	0.20 (0.11, 0.76)	0.123	0.345	0.161	0.079
Oropharyngeal cancer *ACTL6A*	5.02 (2.33, 10.92)	1.49 (0.74, 2.64)	5.49 (1.75, 7.97)	0.123	0.017*	0.674	0.223
CUP *ERCC1*	0.39 (0.23, 0.42)	0.35 (0.02, 0.39)	0.62 (0.27, 0.68)	0.593	0.285	0.285	0.717
CUP *ACTL6A*	4.05 (3.03, 6.36)	1.75 (0.5, 8.84)	3.69 (1.09, 20.88)	0.593	0.285	1.00	0.717
Nasopharyngeal cancer *ERCC1*	0.91 (0.20, 1.16)	0.44 (0.03, 0.63)	0.81 (0.37, 1.17)	0.655	0.180	0.655	0.607
Nasopharyngeal cancer *ERCC1*	7.07 (2.085, 8.51)	6.88 (3.65, 6.67)	9.46 (.86, 13.33)	0.655	0.655	0.655	1.000
Therapy							
CCRT *ERCC1*	0.12 (0.05, 0.39)	0.19 (0.06, 0.29)	0.21 (0.09, 0.51)	0.418	0.010*	0.071	0.003*
CCRT *ACTL6A*	4.77 (1.71, 11.98)	3.25 (0.84, 7.66)	5.33 (1.42, 8.37)	0.053*	0.639	0.135	0.608
Surgery plus adjuvant CCRT *ERCC1*	0.165 (0.05, 0.465)	0.195 (0.057, 0.52)	0.345 (0.080, 1.34)	0.490	0.032*	0.044*	0.027*
Surgery plus adjuvant CCRT *ACTL6A*	4.90 (1.92, 13.59)	5.03 (2.59, 9.26)	7.40 (1.74, 15.84)	0.306	0.661	0.465	0.580
Cycles of chemotherapy							
3 cycles *ERCC1*	0.185 (0.06, 0.38)	0.15 (0.075, 0.87)	0.29 (0.22, 1.88)	0.779	0.025*	0.233	0.093
3 cycles *ACTL6A*	3.89 (0.94, 8.59)	2.57 (0.80, 6.44)	8.75 (1.91, 13.9)	0.401	0.069	0.674	0.417
4 cycles *ERCC1*	0.105 (0.07, 0.28)	0.145 (0.05, 2.83)	0.21 (0.082, 1.85)	0.345	0.6	0.463	0.607
4 cycles *ACTL6A*	1.31 (1.16,12.60)	4.96 (1.59, 16.10)	8.18 (5.86, 9.23)	0.917	0.463	0.345	0.607
5 cycles *ERCC1*	0.14 (0.05, 0.55)	0.19 (0.053, 0.42)	0.29 (0.097, 0.67)	0.741	0.005*	0.083	0.007*
5 cycles *ACTL6A*	4.94 (1.82, 13.2)	4.38 (1.23, 9.36)	5.27 (1.38, 7.66)	0.327	0.462	0.143	0.853
6 cycles *ERCC1*	0.22 (0.03, 0.40)	0.17 (0.06, 0.44)	0.13 (0.07, 1.14)	0.354	0.176	0.212	0.055
6 cycles *ACTL6A*	5.75 (3.38,13.49)	4.25 (0.94,7.49)	5.18 (1.10,15.78)	0.022*	0.711	0.778	0.698

### 5.3 Real-world evidence for *ERCC1*/*ACTL6A* expressions and survival in HNC via meta-analysis

**FIGURE 5:**
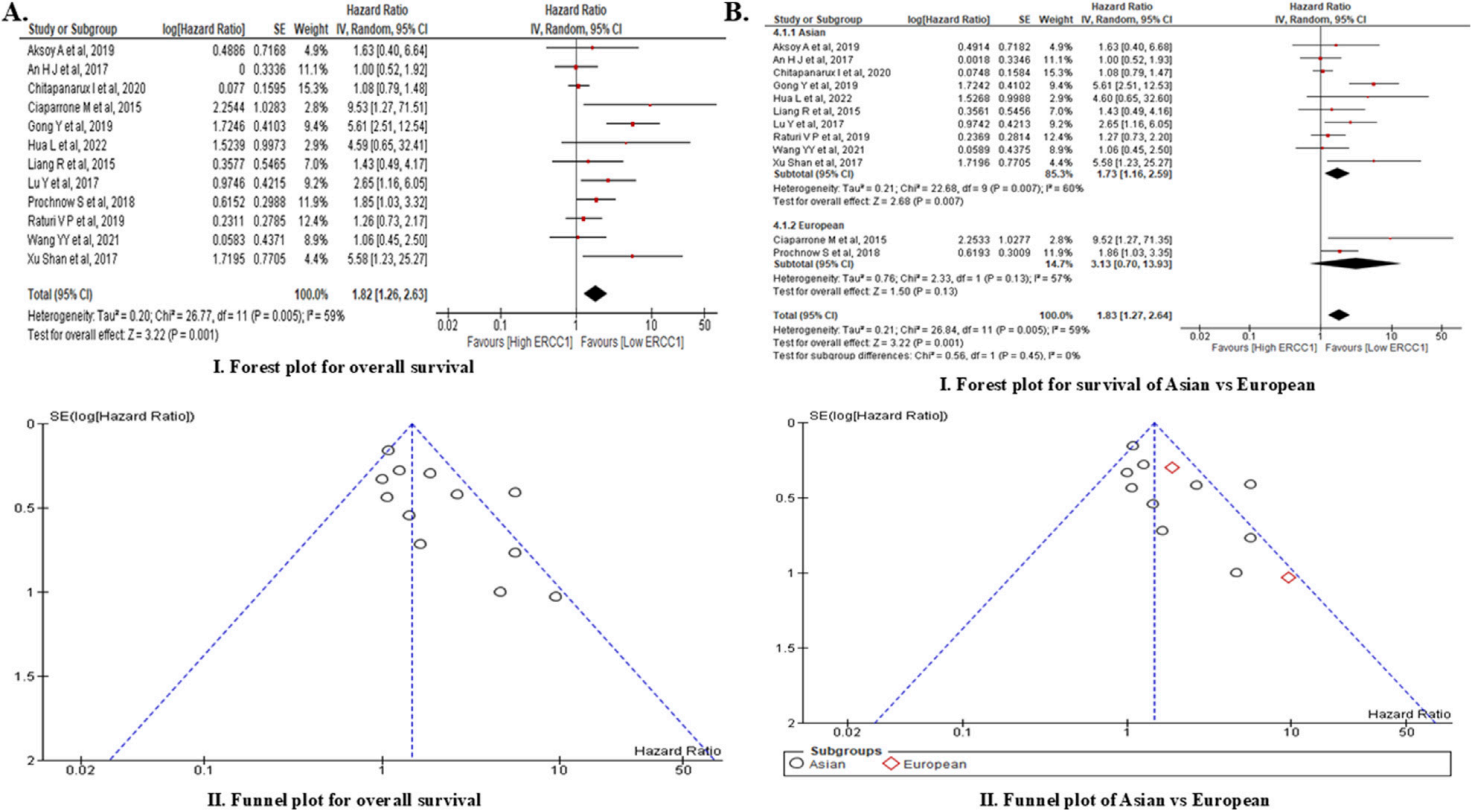
Meta-analysis of *ERCC1* expression and overall survival showing forest plot and funnel plot for **(A)** overall survival of HNC patients and **(B)** comparison of overall survival of Asian vs. European subjects.

A total of 266 articles related to *ERCC1* and HNC were obtained by searching the three databases, of which only 12 articles met the criteria for meta-analysis (Supplementary Figure S6). Out of these 12 studies, only four were conducted prospectively while the remaining eight were conducted retrospectively. The aggregate sample size from all included studies was 2,041, of which 1,810 samples (high *ERCC1* expression: 911 patients, low *ERCC1* expression: 899 patients) were in our analysis (Table 3). Based on the random effects analysis of the pooled data of the 1,810 samples, we found that *ERCC1* expression was linked to poor overall survival among HNC patients, i.e., overexpression of the *ERCC1* gene significantly increased the risk of mortality among HNC patients by 82% (HR: 1.82, 95% CI: 1.26–2.63, *p* = 0.0001) compared to patients who had low expressions of *ERCC1*. However, the analysis showed moderate heterogenicity (*X* ^2^: 26.77, I^2^: 56%, *p* = 0.0005) (Figure 5A). Subgroup analysis of the pooled data also showed that high *ERCC1* expression was significantly linked to poor survival rate among Asians (HR: 1.73, 95% CI: 1.16–2.59, *p* = 0.007) (Figure 5B). Additionally, the funnel plot of the pooled data showed symmetricity with an Egger regression coefficient of −0.152 (*p* = 0.603, 95% CI: −0.783 to 0.479), suggesting no publication bias. A total of 13 articles were identified from the three databases for *ACTL6A* and its association with HNC, of which only four articles were found to have the necessary information; however, none of these articles contained information on *ACTL6A* expression and its impact on survival. Thus, we were unable to conduct a meta-analysis for the *ACTL6A* gene.

**TABLE 3:** Characteristics of all the studies included in the meta-analysis.

S. No.	Author Year	Country Continent	Sample size 2041–231 = 1810	Study Design	Cancer site	Stages	Assay	High vs. low *ERCC1* expression	HR	95% CI	*p*-value	Data extraction model
1	Liang et al. (2015)	China Asia	76 (M:59, F:17)	P	Nasopharynx	III, IVA	IHC	32 (42.1%) vs. 44 (57.89%)	1.43	0.49–4.16	-	KM survival curve
2	Ciaparrone et al. (2015)	Italy Europe	48 (M:39, F:9)	R	Head/Neck	III–IVB	IHC	36 (75%) vs. 12 (25%)	9.53	1.27–71.35	0.028	Multivariate
3	Lu et al. (2017)	China Asia	334 (M:244, F:90)	R	Nasopharynx	I–IVB	IHC	118 (35.32%) vs. 216 (64.7%)	2.65	1.16–6.05	-	KM survival curve
4	Xu et al. (2017)	China Asia	201 (M:132, F:69)	P	Nasopharynx	III–IV	IHC	136 (56.6%) vs. 65 (76.9%)	5.582	1.23–25.27	0.026	Multivariate KM survival curve
5	An et al. (2017)	Korea Asia	204 (M:173, F:31)	R	Head/Neck	I–IV	IHC	136 (66.66%) vs. 68 (33.33%)	1	0.52–1.93	0.99	Multivariate KM survival curve
6	Prochnow et al. (2019)	Germany Europe	453 (159 patients excluded) (M:335, F:118)	R	Head/Neck	I–III	IHC	135 (45.92%) vs. 159 (54.08%)	1.85	1.03–3.35	-	KM survival curve
7	Gong et al. (2019)	China Asia	156 (67 patients excluded) (M:87, F:69)	R	Oral cavity	III, IVA	IHC	41 (22.4%) vs. 48 (84.7%)	5.61	2.51–12.53	-	KM survival curve
8	Raturi et al. (2020)	India Asia	98 (M:98)	P	Larynx	III–IVB	RT-PCR	49 (50%) vs. 49 (50%)	1.26	0.73–2.20	-	KM survival curve
9	Aksoy et al. (2019)	Turkey Asia	33 (5 patients excluded) (M:24, F:9)	R	Nasopharynx	II–IVB	IHC	15 (53.57%) vs. 13 (46.43%)	1.63	0.40–6.68	-	KM survival curve
10	Chitapanarux et al. (2020)	Thailand Asia	262 (M:183, F:79)	R	Nasopharynx	I–IV	IHC	135 (51.52%) vs. 127 (48.48%)	1.08	0.79–1.47	0.647	Multivariate KM survival curve
11	Wang et al. (2021)	Taiwan Asia	98 (M:92, F:6)	R	Oral cavity	I–IV	IHC	58 (59.18%) vs. 40 (40.82%)	1.06	0.45–2.50	0.9	Multivariate KM survival curve
12	Hua et al. (2022)	China Asia	78 (M:59, F:19)	P	Nasopharynx	II	RT-PCR	20 (25.6%) vs. 58 (74.4%)	4.59	0.65–32.60	-	KM survival curve

Note: M: male, F: female, P: prospective, R: retrospective, IHC: immunohistochemistry, RT-PCR: real-time polymerase chain reaction, HR: hazard ratio, KM: Kaplan–Meier. A total of 231 patients were excluded from the analysis because Prochnow et al. and Aksoy et al. did not perform *ERCC1* expression analyses for 159 and 5 patients, respectively, whereas Gong et al. compared *ERCC1* low vs. high for only 89 patients.

## 6 Discussion

**FIGURE 6:**
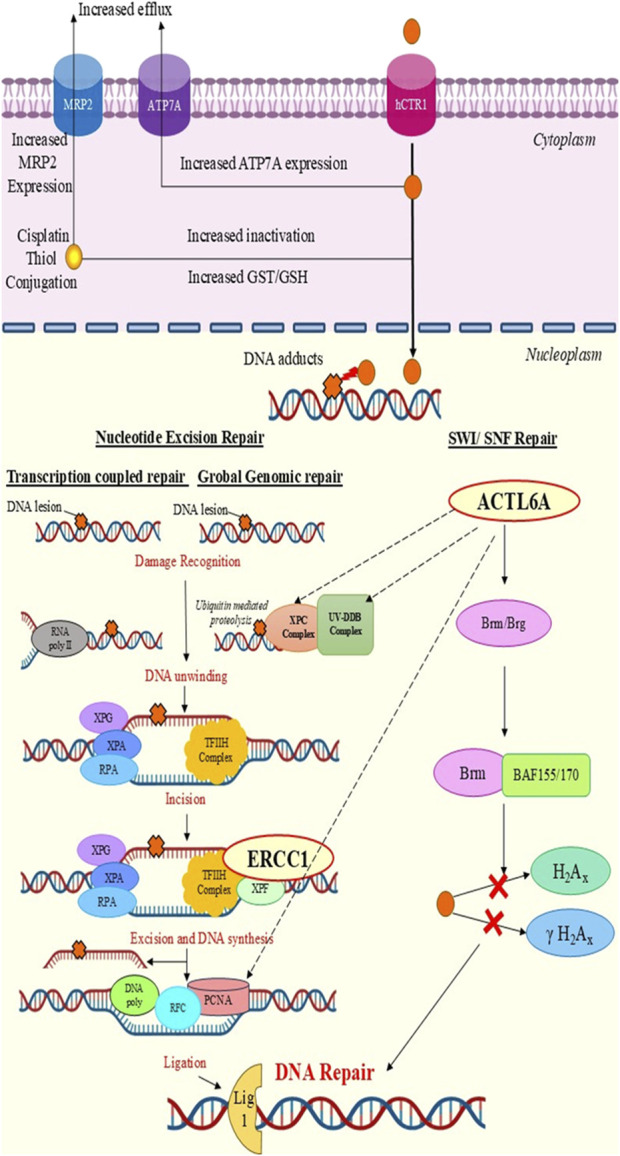
Chemoresistance mechanisms of *ERCC1* and *ACTL6A*. DNA repair is promoted by *ERCC1* via the nucleotide excision repair pathway and by *ACTL6A* through the SWI/SNF complex.

The increases in the median expressions of *ERCC1* and *ACTL6A* before and after CCRT as well as their associations with the poor overall survival outcomes in HNC patients (revealed by integrating computational analysis with meta-analysis) in the present study predict the chemoresistance of genotoxic regimens like cisplatin-based CCRT as these genes are reported to mediate DNA repair via the NER and/or SWI/SNF pathways (Figure 6). Sociodemographically, our findings are consistent with recent epidemiological studies from north India by Badola et al. (2023) and Chauhan et al. (2022), who reported that HNC is more prevalent in men than women, i.e., 87% vs. 13% and 89.4% vs. 10.6%, respectively. Furthermore, Chauhan et al. (2022) and a study on south Indians by SathiyaPriya et al. (2024) observed that nearly half of the study population (48% and 51%, respectively) was aged 40–60 years; in contrast to our study, Badola et al. (2023) and Bagal et al. (2023) found that most of the HNC patients were above 60 years of age followed by those aged 40–60 years. Furthermore, the socioeconomic classes and social habits of the patients in our study resemble those reported by SathiyaPriya et al. (2024), where most of the HNC patients were from the lower middle (62.3%) or lower (37.7%) socioeconomic class and were most commonly associated with tobacco smoking (47.6%) and alcohol consumption (42.4%) followed by tobacco chewing (30.6%) with betel leaf (27.3%) or areca nut (3.3%). Sharp teeth and teeth-mediated injuries to the oral mucosa or tongue have been infrequently linked to cancer of the oral cavity. Lateral tongue carcinoma (odds ratio (OR): 9.1) has been reported as a teeth-mediated injury (Singhvi et al., 2017), while another study reported that lesions due to trauma (OR: 4.5) were observed to be higher among oral cancer patients than lesions in the control group (Piemonte et al., 2018).

Clinically, a significant proportion of the patients in our study were underweight, so we hypothesize that low BMI may be associated with HNC occurrence; this is also supported by the findings from a Korean study, where the incidence of HNC was observed to be higher among underweight individuals (HR: 1.32) than normal weight and overweight patients (HR: 0.89). Furthermore, it was noted that tobacco smoking (HR: 1.448) and alcohol drinking (HR: 1.448) along with low BMI could impose a significantly higher (*p* < 0.05) risk of developing HNC (Kim et al., 2022). A study by Eytan et al. (2019) among 10,524 HNC patients in the United States showed that hypertension, hyperlipidemia, chronic obstructive pulmonary disease, and diabetes were the most common comorbidities at the time of diagnosis, which is consistent with the conditions among our population. Although HNC incidence is not believed to depend on a family history of cancer, we observed that approximately 29.87% of our HNC patients presented with such family history; of these, 16.88% reported a family history of HNC, which is a serious concern. A recent study by Pachuau et al. (2022) on north Indians reporting a family history of cancer among first-degree relatives showed that the risk of developing cancer was significantly higher (OR: 1.921, *p* = 0.037). Furthermore, another study by Li et al. (2021) revealed that the risk of developing HNC among family members increased by 2-fold if the parents/siblings developed HNC. Carcinomas of the oral cavity, larynx, and hypo/oro/nasopharynx were the most predominant types of HNC among our patients, which conform with the sites of HNC development reported from an analysis of 37 Indian cancer registries (Bagal et al., 2023); however, there is a slight disagreement with the findings of Badola et al. (2023) and Chauhan et al. (2022) who reported larynx cancer as the second most-common type after oral cavity cancer. The treatment strategies adopted for our patients (i.e., surgery and CCRT or CCRT alone) comply with the standard treatment guidelines for the management of LAHNC (NCCN Guidelines, 2024; Badola et al., 2023).

Till date, there is only one report of a European study on the dose-dependent expressions of NER genes (Psyrri et al., 2021) among 43 HNC patients, where 35 were responders (81.4%) and 8 were non-responders (18.60%) to cisplatin-based CRT; it was also found that DNA damage, oxidative stress, and NER pathway capacity were significantly higher (*p* < 0.05) in the cisplatin non-responders than responders owing to diminished apoptosis of the tumor cells among the non-responders. This is in agreement with the findings of our study that *ERCC1* expression was significantly increased by 1.64-fold after CCRT compared to the baseline, confirming the increase in NER capacity to clear damaged DNA-cisplatin adducts. Furthermore, approximately 20.78% of the patients in our study showed overexpression of *ERCC1* after 100% CCRT, which is nearly equal to that of the non-responder group reported by Psyrri et al. (2021) *.* Although the DNA repair capacity of *ERCC1* was found increase with therapy, the overall median expression of *ERCC1* was lower than that of the reference gene in our study; this is in agreement with the findings of Psyrri et al. (2021) who observed downregulation of the NER genes, such as *ERCC1*, *ERCC2*/*XPD*, *XPA*, and *XPC*, among HNC patients. Even though we predicted no link between overexpression of *ERCC1* and overall survival via computational analysis, we found that upregulation of *ERCC1* is significantly linked to poor overall survival (HR: 1.82) through the meta-analysis of dose-independent expressions in *ERCC1* studies; this is consistent with the previously reported HRs (2.14 and 1.95) among *ERCC1* overexpressing HNC patients (Xuelei et al., 2015; Bišof et al., 2016). These findings are attributed to the increased NER capacity via *ERCC1*, which may be associated with CCRT resistance and poor clinical outcomes among HNC patients. Furthermore, nearly half of the HNC patients (50.33%) among the studies included in the meta-analysis showed high *ERCC1* expressions, which is comparatively higher than that observed in our study where 14.29% and 20.78% of the patients had high expressions at baseline and after 100% CCRT, respectively. The details of the studies included in the meta-analysis are outlined in Table 3 (Prochnow et al., 2019; Liang et al., 2015; Ciaparrone et al., 2015; Lu et al., 2017; Xu et al., 2017; An et al., 2017; Gong et al., 2019; Aksoy et al., 2019; Raturi et al., 2020; Chitapanarux et al., 2020; Wang et al., 2021; Hua et al., 2022).

Presently, there are no available studies on evaluating the dose-dependent expression of *ACTL6A*. However, *ACTL6A* has been applauded as a novel gene responsible for cisplatin resistance in various cancers, such as ovarian, lung, and esophageal cancers (Xiao et al., 2021). Overexpression of *ACTL6A* is believed to mediate DNA repair via the SWI/SNF complex by regulating the expression of the Brahma related gene 1 (*Brg1*) or Brahma (*Brm*) and promoting its binding to BRAF155/BRAF170 to hinder cisplatin-mediated H2AX or γH2AX activation (Xiao et al., 2021). Out of the four documents that we retrieved through a systematic search, three studies used human tissue samples to explore *ACTL6A* as a biomarker for cell proliferation, invasion, or metastasis, leading to unfavorable/poor prognosis among HNC patients (Xiao et al., 2021; Liu et al., 2024; Dang et al., 2020; Saladi et al., 2017). A recently published Chinese study by Liu et al. (2024) reported that *ACTL6A* is significantly overexpressed in oral cancer tissues compared to normal tissues and proposed that tumor factors like E2F7, TP63, and microRNA has-mir-381 regulate *ACTL6A* expression to promote cell proliferation, migration, and invasion through the WNT and TP53 signaling pathways. It has also been reported that high *ACTL6A* expression is significantly linked to TP53 mutation rate, which could contribute to chemoresistance to CRT (Xiao et al., 2021). Similarly, studies by Dang et al. (2020) and Saladi et al. (2017) confirmed overexpression of *ACTL6A* in HNC, anticipating that *ACTL6A* interacts with P63 and activates the Yes-associated protein (YAP); this could lead to translocation of YAP into the nucleus, which promotes tumorigenesis via the Hippo-YAP signaling pathway (Dang et al., 2020; Saladi et al., 2017). These findings are correlated with those of our study, where we predicted and demonstrated *ACTL6A* overexpression in HNC via computational analysis and qPCR across the therapy. Furthermore, overexpression of *ACTL6A* was also predicted to be a significant contributor to poor overall survival. However, none of these studies have demonstrated the involvement of *ACTL6A* in DNA repair in HNC or its relation to NER. The present study indicates that *ACTL6A* interacts with the UV-DDB complex, XPC complex of GGR-NER, and PCNA of TCR-NER, thereby contributing to DNA repair. We also found significant and marginally significant correlations between *ERCC1* and *ACTL6A* expressions after 50% (*p* = 0.003) and 100% (*p* = 0.06) CCRT, respectively, among the HNC patients, which supports the hypothesis of ACTL6A-mediated NER activation.

Immune cell infiltration of the tumor cells and their interactions with the tumor microenvironment have been proposed to modulate the immune cells, leading to immunosuppression and chemoresistance, thereby resulting in poor clinical outcomes like metastasis and poor survival (Wondergem et al., 2020; Jumaniyazova et al., 2022). However, the inconsistencies in these findings pose conflicts for acceptability in clinical practice. Neutrophil-infiltrating tumor cells undergo polarization to form two phenotypes N1 and N2 that exbibit antitumor and protumor properties, respectively. Here, the N2 phenotype makes the tumor more aggressive by inducing genetic instabilities, angiogenesis, metastasis, and immunosuppression (Wondergem et al., 2020; Jumaniyazova et al., 2022). However, infiltration of the tumor cells by myeloid dendritic cells was reported to exert antitumor and anti-inflammatory effects via increased tumor leucocyte infiltration, whereas plasmacytoid dendritic cell infiltration was reported to be linked with unfavorable outcomes (Wondergem et al., 2020; Jumaniyazova et al., 2022). Similar to neutrophils, macrophages also polarize into M1 and M2 phenotypes, of which the M2 phenotype is linked with protumoral activities, such as tumor migration, invasion, metastasis, and poor survival (Wondergem et al., 2020; Jumaniyazova et al., 2022). To some extent, CD8^+^ infiltration has been reported to be associated with favorable outcomes, whereas the effects of CD4^+^ are yet to be clarified (Wondergem et al., 2020; Jumaniyazova et al., 2022). These findings may be important in chemoresistance as both *ERCC1* and *ACTL6A* expressions were found to increase the infiltration of immune cells, such as CD4^+^ cells, macrophages, myeloid dendritic cells, and B cells.

Nevertheless, knockdown of DNA repair expression could reverse the chemoresistance of or restore sensitivity to the cisplatin or platinum drugs. Among the HNC patients with cisplatin-based CRT resistance or platinum drug resistance, FDA-approved drugs like cyclosporin, 5-fluorouracil, doxorubicin, gemcitabine, paclitaxel, thalidomide, and panobinostat can be repurposed to downregulate *ERCC1* and *ACTL6A* genes. Although paclitaxel and 5-fluorouracil are used for the management of HNC (NCCN Guidelines, 2024), there are no data regarding the use of these anticancer agents against *ERCC1* and *ACTL6A* genes among HNC patients. Thus, we recommend the clinical investigation of these anticancer agents in combination with platinum therapy to mitigate platinum drug resistance or achieve better efficacy of CCRT among HNC patients. Moreover, E-X PPI2, E-X AS7, and panobinostat (a HDAC inhibitor) have been reported to silence *ERCC1* and *ACTL6A* expressions in melanoma and ovarian/lung cancers, respectively, via *in vitro* and preclinical experiments (Xiao et al., 2021; McNeil et al., 2015). Similarly, siRNA- and shRNA-transfected HNC cell lines have shown promising results for downregulating *ACTL6A* expressions (Liu et al., 2024; Dang et al., 2020; Saladi et al., 2017); these findings offer hope for tackling chemoresistance in cancer therapy.

## 7 Limitations and future directions

Although the present study was conducted with a unique methodology to decipher the dose-dependent expressions of chemoresistance genes and has the advantage of a molecularly sensitive technique like qPCR compared to IHC, we were unable to evaluate the tumor burden via the RECIST criteria, which should be addressed in the future to generalize our findings. However, the findings of the current study can also be utilized to conduct a novel clinical trial to investigate the dose-dependent expressions of *ERCC1* and *ACTL6A* among large HNC cohorts along with RECIST mapping of the tumor burden for clinical applicability. Furthermore, *ACTL6A* (Liu et al., 2024; Dang et al., 2020; Saladi et al., 2017) and *ERCC1* (Seetharam et al., 2010; Wang et al., 2017) can be targeted using siRNA and shRNA to silence their expressions to counteract chemoresistance. The present study also offers a hypothesis regarding the associations between chromatin remodeling genes and their DNA repair capacities via the SWI/SNF as well as NER pathways, which could motivate future research in this field.

## 8 Conclusion

We demonstrate that increased expressions of *ERCC1* and *ACTL6A* during and/or after cisplatin-based CRT can mediate DNA repair, leading to chemoresistance in HNC as well as poor overall survival thereof. *ERCC1* and *ACTL6A* are known to regulate several repair pathways that participate in DNA repair processes. *ACTL6A* is also known to promote DNA repair activity by interacting with the UV-DDB complex, XPC complex of GGR-NER, and PCNA of TCR-NER. Thus, *ERCC1* and *ACTL6A* are critical evolutionarily conserved core proteins with theranostic potential for cisplatin or cisplatin-based CRT resistance that can be detected via liquid biopsy. Furthermore, repurposing some of the available FDA-approved drugs for targeting *ERCC1* and *ACTL6A* is proposed as a novel approach to counteract chemoresistance in clinical practice.

## Data Availability

The original contributions presented in the study are included in the article/Supplementary Material, further inquiries can be directed to the corresponding author.

## Abbreviations

DNA: deoxyribonucleic acid, HNC: head and neck cancer, ERCC1: excision repair cross-complementation group 1, ACTL6A: actin-like protein 6A, CRT: chemoradiotherapy, FDA: Food and Drug Administration, NER: nucleotide excision repair, SWI/SNF: switch/sucrose non-fermenting, LAHNC: locally advanced head and neck cancer, CCRT: concurrent chemoradiotherapy, R/MHNC: recurrent/metastatic head and neck cancer, HR: hazard ratio, TCR: transmission-coupled repair, GGR: global genome repair, PPI: protein–protein interaction, BP: biological process, CC: cellular component, MF: molecular function, IHC: immunohistochemistry, BMI: body mass index, OR: odds ratio.
