# Supplementary material for: A multimodal approach for establishing ACTL6A and ERCC1 as chemoresistance genes in locally advanced head and neck cancer
Source: Front Pharmacol. 2025 May 29;16:1541987. doi: 10.3389/fphar.2025.1541987 (PMC12159016; doi:10.3389/fphar.2025.1541987)
Supplement: Supplementary file 1 [file DataSheet1.docx]

**Supplementary file**

**Materials and Methods**

**4.2 Search Strategy**

**1. PUBMED**

| **Search area** | **Search terms** | **Result count** |
| --- | --- | --- |
| #1 ERCC1 | "ERCC1"[Title/Abstract] OR "excision repair cross complementing group 1"[Title/Abstract] OR "excision repair cross complementation group 1"[Title/Abstract] | 2602 |
| #2 ACTL6A | "ACTL6A"[Title/Abstract] OR "Actin-like protein 6A"[Title/Abstract] | 99 |
| Location of cancer  #3 | "Head and Neck"[Title/Abstract] OR "Head"[Title/Abstract] OR "Neck"[Title/Abstract] OR "upper aerodigestive tract"[Title/Abstract] OR "UADT"[Title/Abstract] OR "Lip"[Title/Abstract] OR "Tongue"[Title/Abstract] OR "Gingival"[Title/Abstract] OR "gingiva"[Title/Abstract] OR "Mouth"[Title/Abstract] OR "Oral"[Title/Abstract] OR "Palatal"[Title/Abstract] OR "Jaw"[Title/Abstract] OR "Parotid"[Title/Abstract] OR "Salivary"[Title/Abstract] OR "Salivary gland"[Title/Abstract] OR "Sublingual gland"[Title/Abstract] OR "Submandibular gland"[Title/Abstract] OR "Tonsil"[Title/Abstract] OR "Tonsillar"[Title/Abstract] OR "Oropharyngeal"[Title/Abstract] OR "Oropharynx"[Title/Abstract] OR "Nasopharynx"[Title/Abstract] OR "Nasopharyngeal"[Title/Abstract] OR "piriform sinus"[Title/Abstract] OR "piriform fossa"[Title/Abstract] OR "Hypopharyngeal"[Title/Abstract] OR "Pharyngeal"[Title/Abstract] OR "Pharynx"[Title/Abstract] OR "Laryngeal"[Title/Abstract] OR "Larynx"[Title/Abstract] OR "paranasal sinus"[Title/Abstract] OR "nasal*"[Title/Abstract] OR "Buccal"[Title/Abstract] OR "glottis"[Title/Abstract] OR "Nose"[Title/Abstract] OR "Subglottic"[Title/Abstract] | 1773196 |
| Cancer  #4 | "cancer*"[Title/Abstract] OR "carcinoma*"[Title/Abstract] OR "adenocarcinoma*"[Title/Abstract] OR "malignan*"[Title/Abstract] OR "tumor*"[Title/Abstract] OR "tumour*"[Title/Abstract] OR "neoplasm*"[Title/Abstract] | 4024439 |
| Time filter | #1 AND #3 AND #4 AND (Time filter: 2015/1/1:2023/8/31[pdat]) | 69 |
|  | #2 AND #3 AND #4 (Time filter: Till 26^th^ June 2024) | 4 |

**2. SCOPUS**

| **Search area** | **Search terms** | **Results count** |
| --- | --- | --- |
| Gene  #1 | TITLE-ABS-KEY ("ERCC1") OR TITLE-ABS-KEY ("excision repair cross complementing group 1") OR TITLE-ABS-KEY ("excision repair cross complementation group 1") | 2902 |
| #2 | TITLE-ABS-KEY (“ACTL6A") OR TITLE-ABS-KEY ("Actin-like protein 6A") | 144 |
| Location of cancer  #3 | TITLE-ABS-KEY ("Head and Neck") OR TITLE-ABS-KEY("Head") OR TITLE-ABS-KEY ("Neck") OR TITLE-ABS-KEY ("upper aerodigestive tract") OR TITLE-ABS-KEY ("UADT") OR TITLE-ABS-KEY ("Lip") OR TITLE-ABS-KEY ("Tongue") OR TITLE-ABS-KEY ("Gingival") OR TITLE-ABS-KEY ("gingiva") OR TITLE-ABS-KEY ("Mouth") OR TITLE-ABS-KEY ("Oral") OR TITLE-ABS-KEY ("Palatal") OR TITLE-ABS-KEY ("Jaw") OR TITLE-ABS-KEY ("Parotid") OR TITLE-ABS-KEY ("Salivary") OR TITLE-ABS-KEY ("Salivary gland") OR TITLE-ABS-KEY ("Sublingual gland") OR TITLE-ABS-KEY ("Submandibular gland") OR TITLE-ABS-KEY ("Tonsil") OR TITLE-ABS-KEY ("Tonsillar") OR TITLE-ABS-KEY ("Oropharyngeal") OR TITLE-ABS-KEY ("Oropharynx") OR TITLE-ABS-KEY("Nasopharynx") OR TITLE-ABS-KEY ("Nasopharyngeal") OR TITLE-ABS-KEY ("piriform sinus") OR TITLE-ABS-KEY ("piriform fossa") OR TITLE-ABS-KEY ("Hypopharyngeal") OR TITLE-ABS-KEY ("Pharyngeal") OR TITLE-ABS-KEY("Pharynx") OR TITLE-ABS-KEY ("Laryngeal") OR TITLE-ABS-KEY ("Larynx") OR TITLE-ABS-KEY ("paranasal sinus") OR TITLE-ABS-KEY ("nasal*") OR TITLE-ABS-KEY ("Buccal") OR TITLE-ABS-KEY ("glottis") OR TITLE-ABS-KEY ("Nose") OR TITLE-ABS-KEY ("Subglottic") | 3,471,704 |
| Cancer  #4 | TITLE-ABS-KEY("cancer*") OR TITLE-ABS-KEY ("carcinoma*") OR TITLE-ABS-KEY ("adenocarcinoma*") OR TITLE-ABS-KEY("malignan*") OR TITLE-ABS-KEY("tumor*") OR TITLE-ABS-KEY ("tumour*") OR TITLE-ABS-KEY("neoplasm*") | 6,230,096 |
|  | #1 AND #3 AND #4 | 189 |
| Time filter | #1 AND #3 AND #4 AND (Time filter: 2015:2023[pdat]) | 84 |
|  | #2 AND # 3 AND #4 AND (Time filter: 26^th^ June 2024) | 6 |

**3. web of science**

| **Search area** | **Search terms** | **Results count** |
| --- | --- | --- |
| #1 ERCC1 | ALL=(("ERCC1" OR "excision repair cross complementing group 1" OR "excision repair cross complementation group 1")) | 3169 |
| #2 ACTL6A | ALL= ("ACTL6A"[Title/Abstract] OR "Actin-like protein 6A"[Title/Abstract] | 111 |
| Location of cancer  #2 | ALL=(("Head and Neck" OR "Head" OR "Neck" OR “upper aerodigestive tract" OR "UADT" OR "Lip" OR "Tongue" OR "Gingival" OR "gingiva" OR "Mouth" OR "Oral" OR "Palatal" OR "Jaw" OR "Parotid" OR "Salivary" OR "Salivary gland" OR "Sublingual gland" OR "Submandibular gland" OR "Tonsil" OR "Tonsillar" OR "Oropharyngeal" OR "Oropharynx" OR "Nasopharynx" OR "Nasopharyngeal" OR "piriform sinus" OR "piriform fossa" OR "Hypopharyngeal" OR "Pharyngeal" OR "Pharynx" OR "Laryngeal" OR "Larynx" OR "paranasal sinus" OR "nasal*" OR "Buccal" OR "glottis" OR "Nose" OR "Subglottic")) | 1960960 |
| #3 Cancer | ALL=(("cancer*" OR "carcinoma*" OR "adenocarcinoma*" OR "malignan*" OR "tumor*" OR ("tumour*") OR "neoplasm*")) | 4392584 |
|  | #1 AND #3 AND #4 | 260 |
| Time filter | #1 AND #3 AND #4 AND (Timer filter: 2015-01-01 to 2023-08-31) | 113 |
|  | #2 AND # 3 AND #4 AND (Time filter: 26^th^ June 2024) | 4 |

**ERCC1:**

**Total (PubMed, Scopus, Web of Science)** = 69+84+113 = 266 articles

**Duplicates Removed:** 131 articles

**Title Abstract/ Screening**: Excluded: 113 articles, Included: 22 articles

**Full text screening:** Excluded: 10 articles, Included: 12 articles

**ACTL6A:**

**Total (PubMed, Scopus, Web of Science)** = 4+6+4 = 14 articles

**Duplicates Removed:** 8 articles

**Title Abstract/ Screening**: Excluded: 1 article, Included: 4 articles

**Full text screening:** Excluded: 1 article, Included: 3 articles

**5 Results**

- 1. **Computational Analysis**

***5.1.2 mRNA and Tissue Expression of ERCC1 and ACTL6A in HNC***

**Table ST 1: Median mRNA Expression of ERCC1 and ACTL6A in cancer and normal tissue**

| **Parameters** | **ERCC1 Expression** | | **ACTL6A Expression** | |
| --- | --- | --- | --- | --- |
|  | **cancer** | **Normal** | **Cancer** | **Normal** |
| **Stages** | | | |  |
| Stages I | 58.34 | 46.25 | 61.26 | 30.82 |
| Stage II | 63.34 |  | 71.75 |  |
| Stage III | 61.45 |  | 67.07 |  |
| Stage IV | 65.12 |  | 72.09 |  |
| **Race** | | | |  |
| Caucasian | 64.33 | 46.25 | 71.75 | 30.82 |
| African-American | 65.16 |  | 71.39 |  |
| Asian | 58.80 |  | 65.22 |  |
| **Age Groups** | | | |  |
| 21-40 years | 51.94 | 46.25 | 65.22 | 30.82 |
| 41-60 years | 63.83 |  | 79.07 |  |
| 61-80 years | 67.07 |  | 70.44 |  |
| 81-100 years | 63.46 |  | 46.35 |  |
| **Tumor Grade** |  |  |  |  |
| Grade I | 59.75 | 46.25 | 47.81 | 30.82 |
| Grade II | 64.84 |  | 73.67 |  |
| Grade III | 65.42 |  | 75.17 |  |
| Grade IV | 61.50 |  | 127.26 |  |

| 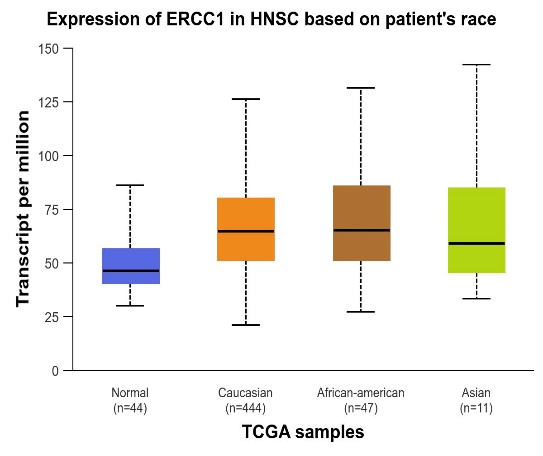 | 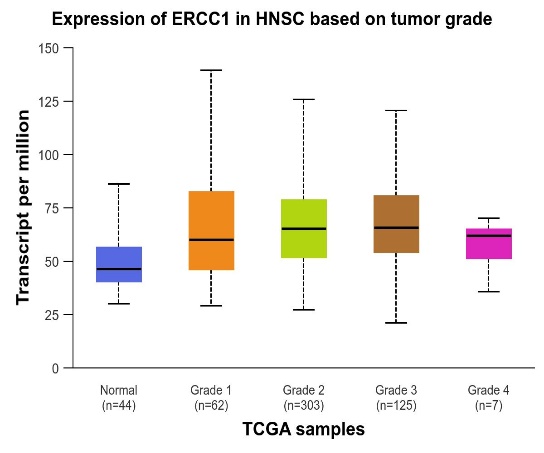 | 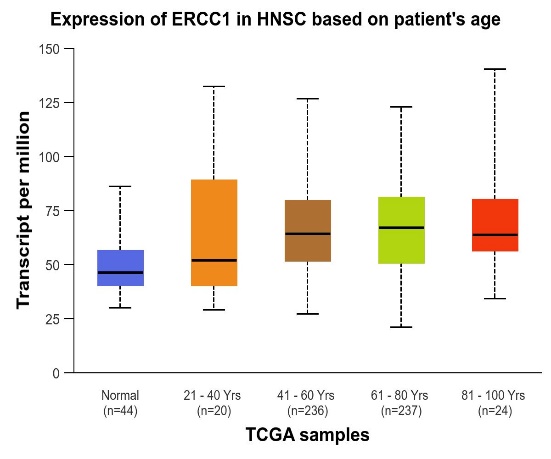 |
| --- | --- | --- |
| 1. **Racial origin** | **b. Tumor Grade** | **c. Age Groups** |
| **mRNA expression of ERCC1** | | |
| 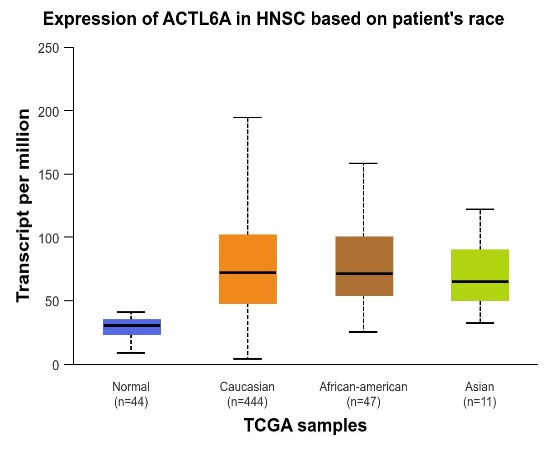 | 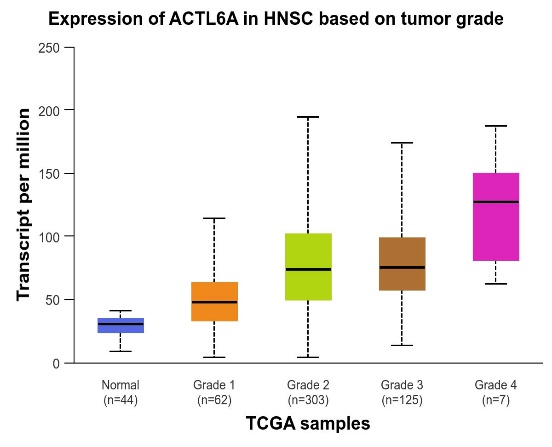 | 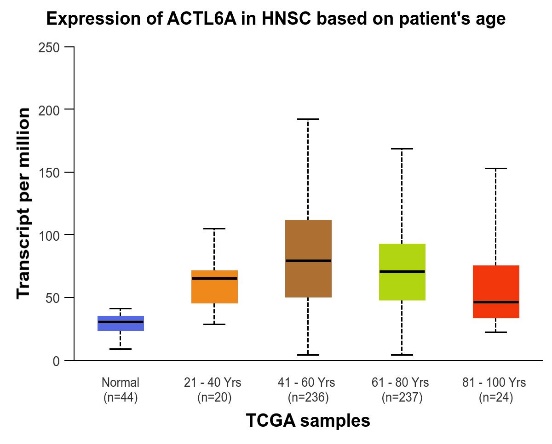 |
| 1. **Racial origin** | 1. **Tumor Grade** | 1. **Age Groups** |
| **mRNA expression of ACTL6A** | | |
| **Figure SF 1: mRNA expression of ERCC1 and ACTL6A** | | |

| **ERCC1 Expression** | | **ACTL6A Expression** | |
| --- | --- | --- | --- |
| 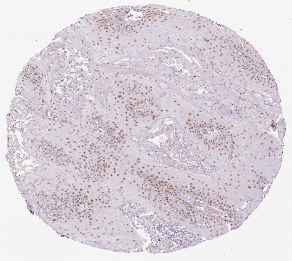 | 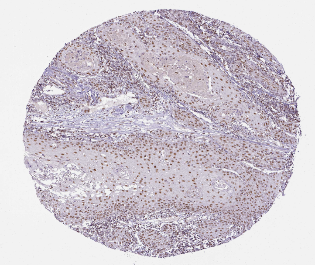 | 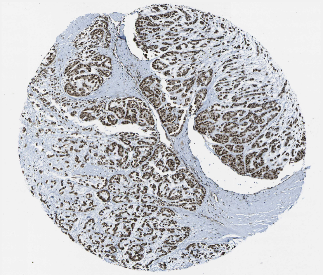 | 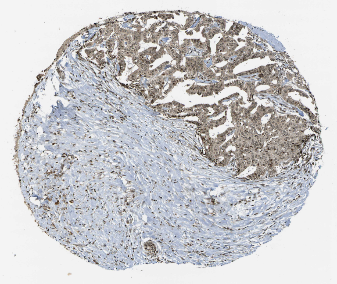 |
| **CAB072859**  Moderate Nuclear Staining | **CAB072860**  Moderate Nuclear Staining | **CAB012188**  Strong Cytoplasmic/ membranous nuclear staining | **CAB012188**  Moderate Cytoplasmic/ membranous nuclear staining |

**Figure SF2: Immunohistochemistry of ERCC1 and ACTL6A**

**Table ST2: Top Five Mutations leading to ERCC1 overexpression**

| **Mutation of** | **Mean expression (mutant)** | **Mean expression (wild)** | **No. of Mutant** | **Number of wild** | **FC (mutant/wild)** | **Direction** | **p-value** |
| --- | --- | --- | --- | --- | --- | --- | --- |
| CENPF | 2319.2 | 1410.55 | 10 | 484 | 1.64 | up | 0.0002 |
| KMT2B | 2109.19 | 1406.17 | 16 | 478 | 1.5 | up | 0.000323 |
| TSHZ3 | 2300.31 | 1405.39 | 13 | 481 | 1.64 | up | 0.000551 |
| DVL1 | 2445.4 | 1418.55 | 5 | 489 | 1.72 | up | 0.000762 |
| CASK | 2167 | 1421.39 | 5 | 489 | 1.52 | up | 0.00166 |

| 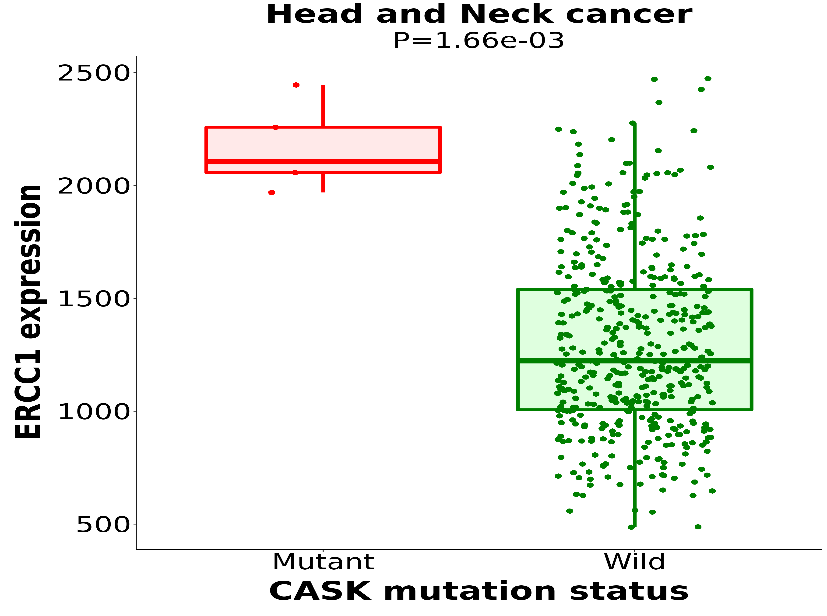 | 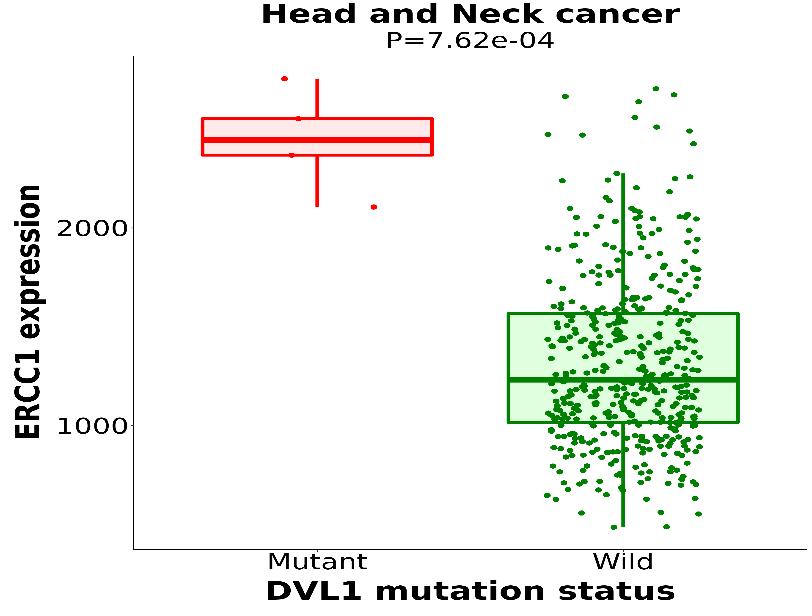 |
| --- | --- |
| **Figure SF3: Mutation influencing ERCC1 expression** | |

**Table ST3: Top five Mutations leading to ACTL6A overexpression**

| **Mutation of** | **Mean expression (mutant)** | **Mean expression (wild)** | **No. of Mutant** | **Number of wild** | **FC (mutant/wild)** | **Direction** | **p-value** |
| --- | --- | --- | --- | --- | --- | --- | --- |
| FBN1 | 3702.07 | 2323.72 | 15 | 479 | 1.59 | up | 0.000172 |
| STEAP4 | 4378.09 | 2319.74 | 11 | 483 | 1.89 | up | 0.000322 |
| SCN8A | 4472.62 | 2330.89 | 8 | 486 | 1.92 | up | 0.00107 |
| OR8H2 | 4314.88 | 2333.48 | 8 | 486 | 1.85 | up | 0.0014 |
| CASZ1 | 3603.18 | 2337.39 | 11 | 483 | 1.54 | up | 0.00197 |

| 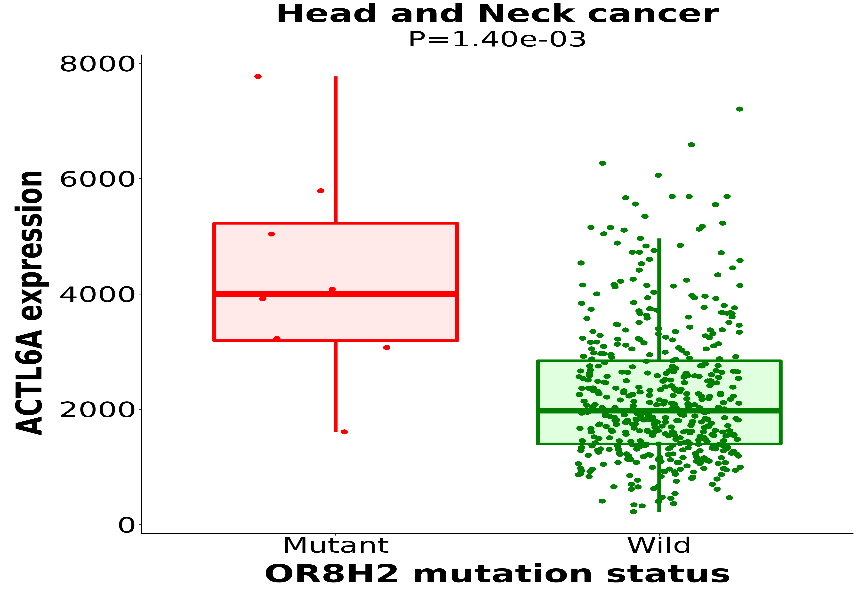 | 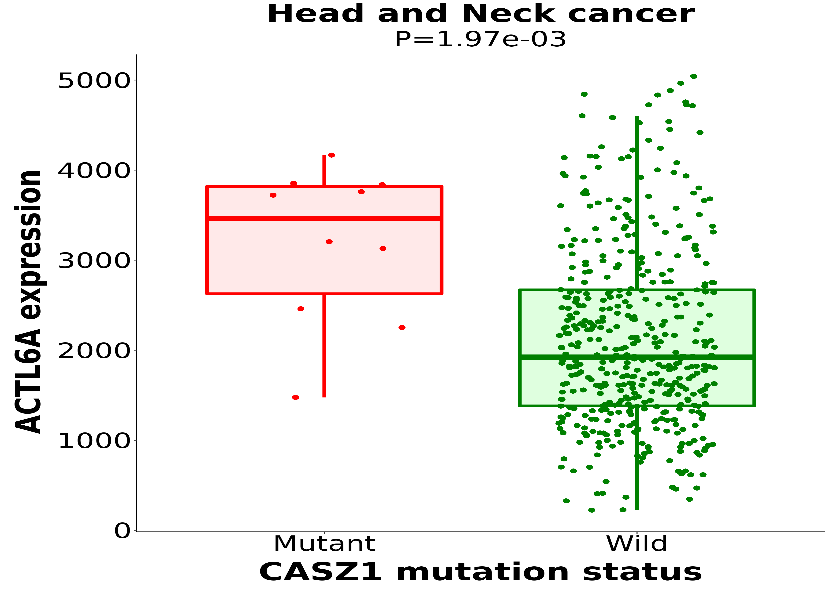 |
| --- | --- |
| **Figure SF4: Mutation influencing ACTL6A expression** | |

**5.1.3 *Impact of ERCC1 and ACTL6A expression on tumor cell infiltration and survival in HNC***

| 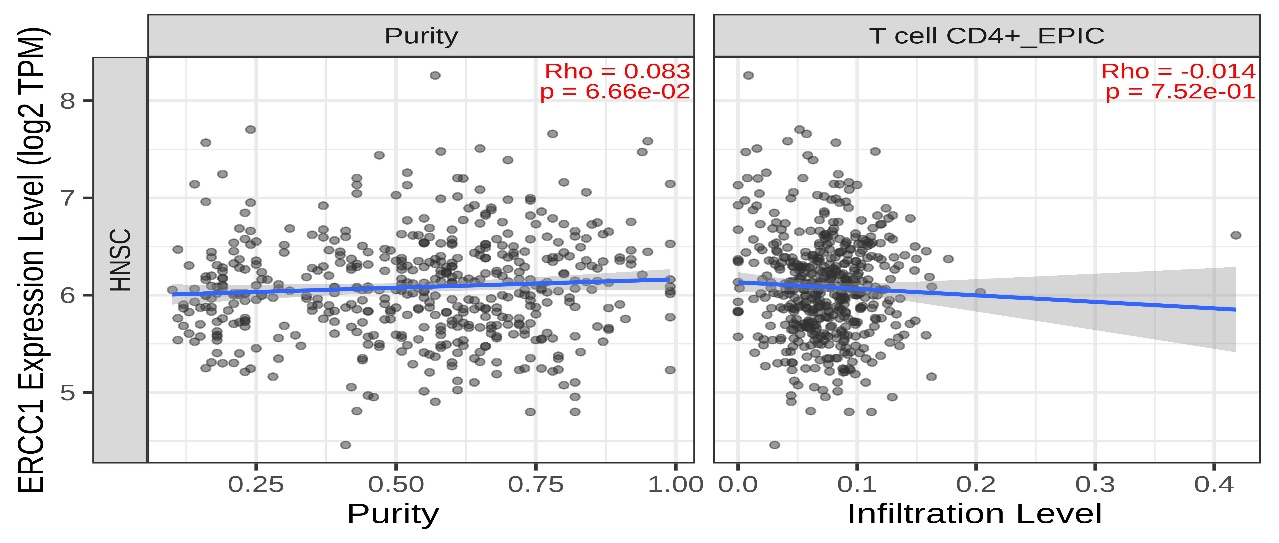 | 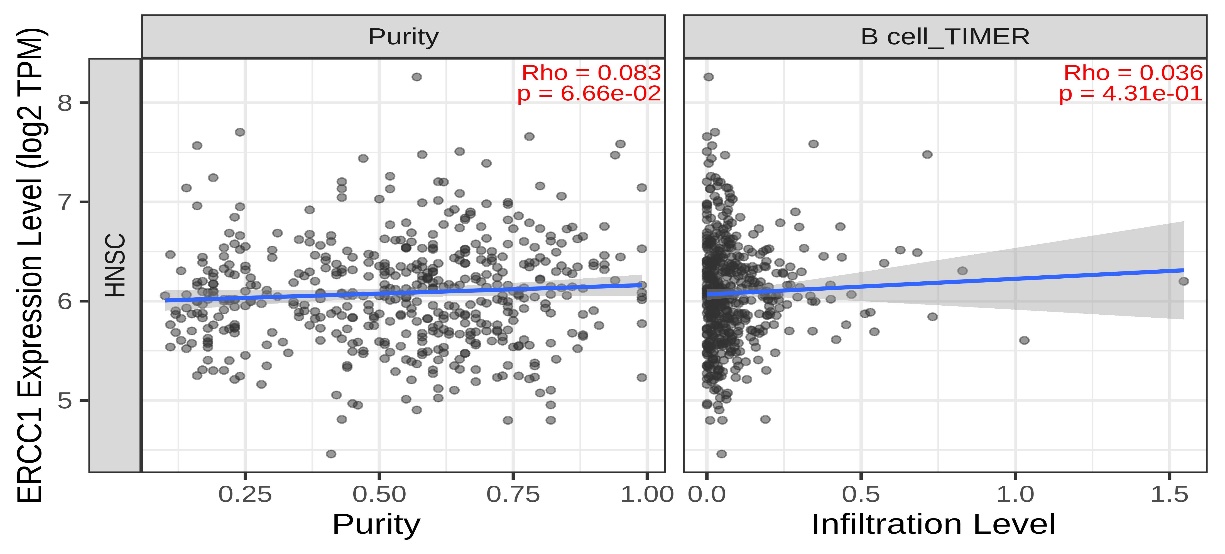 | 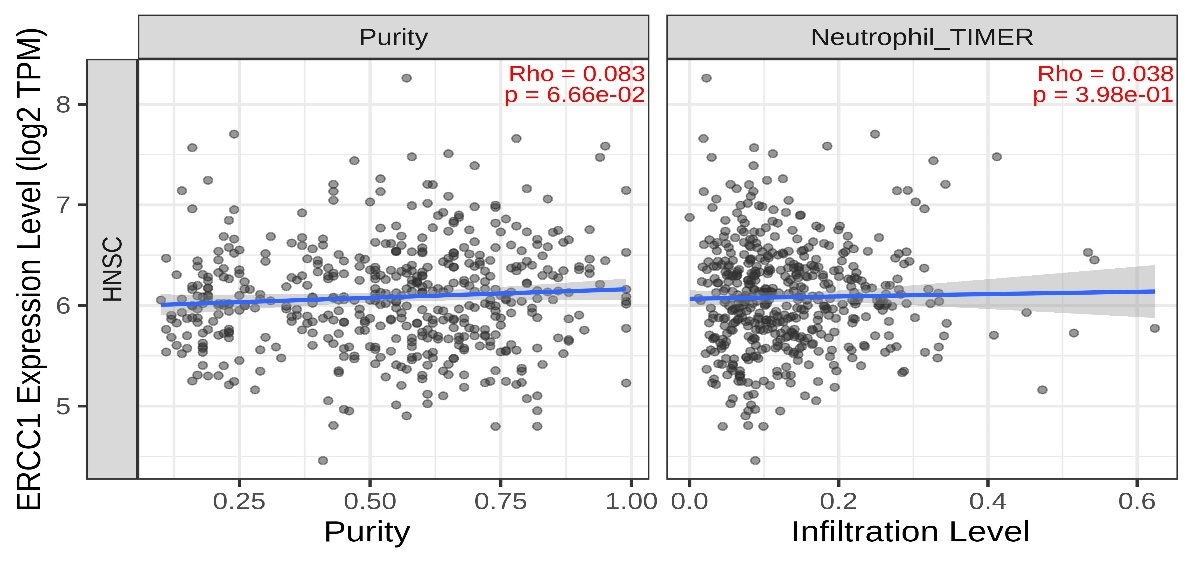 |
| --- | --- | --- |
| **CD4+** | **B-cell** | **Neutrophil** |
| 1. **ERCC1 expression and Immune cell infiltration** | | |
| 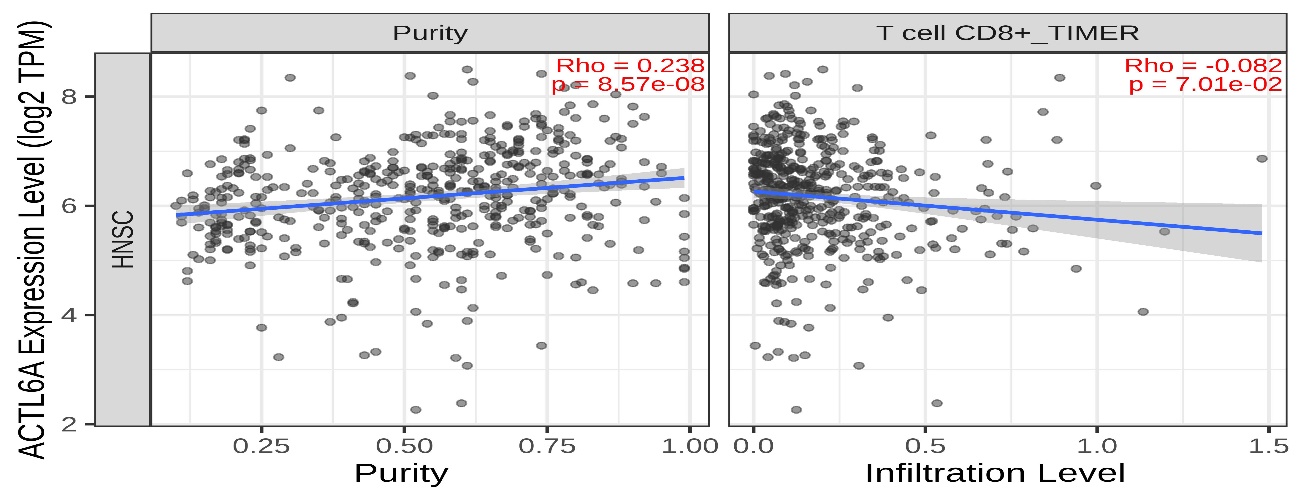 | 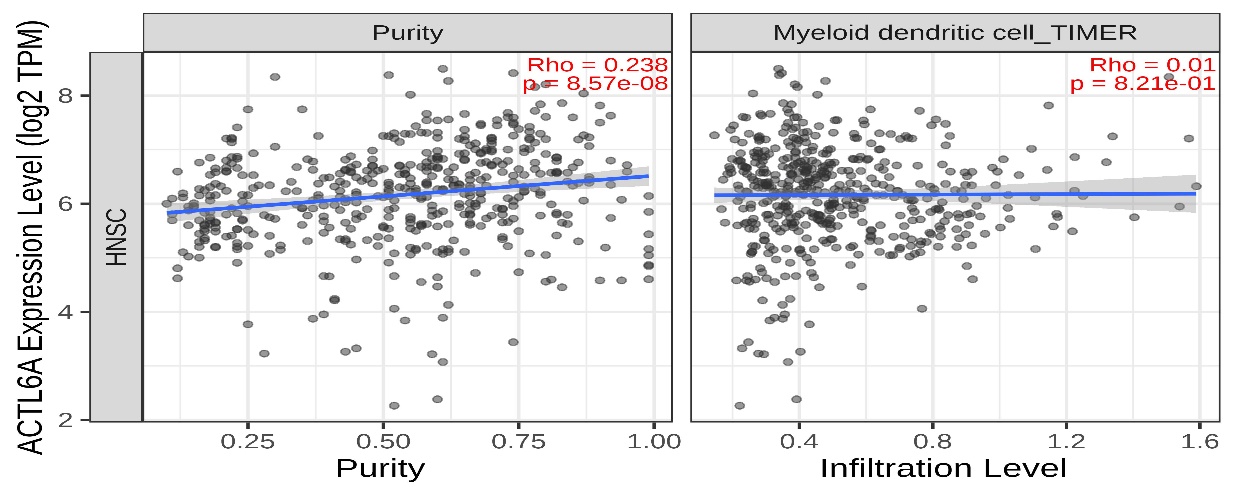 | 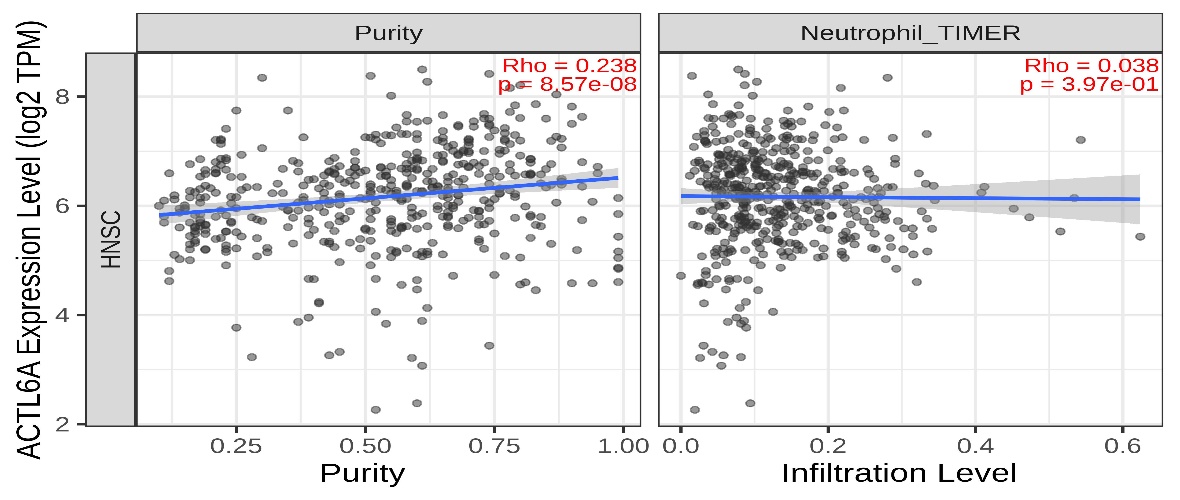 |
| **CD8+** | **Myeloid dendritic cell** | **Neutrophil** |
| 1. **ACTL6A expression and Immune cell infiltration** | | |
| **Figure SF5: ERCC1/ACTL6A expression and immune infiltration** | | |

- - 1. ***Potential drug candidates and their binding affinity with ERCC1 and ERCC1***

**Table ST4: Molecular docking of FDA approved drug candidates with ERCC1 and ACTL6A**

| **Gene** | **Drugs** | **Interaction Score by DGIbd** | **Binding**  **Energy** | **Amino-acid Residue** |
| --- | --- | --- | --- | --- |
| **ERCC1** | **FDA approved Drugs** | | | |
|  | Cyclosporin | 0.10 | — | — |
|  | Fluorouracil | 0.074 | -1.412 | LYS162, ALA192 |
|  | Doxorubicin | 0.088 | — | — |
|  | Gemcitabine | 0.047 | -3.707 | LEU157, GLN 158, LYS162, ALA192 |
|  | Paclitaxel | 0.040 | — | — |
|  | Thalidomide | 0.237 | -1.409 | ASN163 |
|  | **Non-FDA approved drugs** | | | |
|  | Staurosporine | 0.257 | — | — |
|  | Herbimycin A | 1.094 | — | — |
| **ACTL6A** | **FDA approved Drugs** | | | |
|  | Panobinostat | 0.398 | -4.198 | ARG24, SER123, ASP171 |
|  | **Non-FDA approved drugs** | | | |
|  | Sphingosine 1-Phosphate | 1.459 | -3.119 | ASP17, ARG24, ASP171, GLY353 |
|  | Sphingosylphosphorylcholine | 13.127 | — | — |
| **Note:** All the binding energy are in Kcal/mol. **“—" :** No Docking Result obtained | | | | |

- 1. **Human Experimentation**

**Table ST5: Expression pattern of ERCC1 and ACTL6A across therapy among HNC patients**

| **Therapy** | **ERCC1 Expression** | | **ACTL6A Expression** | |
| --- | --- | --- | --- | --- |
|  | **Up** | **Down** | **Up** | **Down** |
| Before CCRT | 11 (14.29%) | 66 (85.71%) | 68 (88.31%) | 9 (11.69%) |
| After 50% CCRT | 7 (9.09%) | 70 (90.91%) | 58 (75.32%) | 19 (24.68%) |
| After 100% CCRT | 16 (20.78%) | 61 (79.22%) | 65 (84.42%) | 12 (15.58%) |
| Baseline to 1^st^ follow up | 46 (59.74%) | 31 (40.26%) | 44 (57.14%) | 33 (42.86%) |
| Baseline to 2^nd^ follow up | 52 (67.53%) | 25 (32.47%) | 37 (48.05%) | 40 (51.95%) |
| 1^st^ to 2^nd^ follow up | 56 (72.73%) | 21 (27.27%) | 39 (50.65%) | 1. (49.35%) |

- 1. ***Real-world evidence for ERCC1/ACTL6A expression and survival among HNC via Meta-analysis***

**Identification of studies via databases**

Records removed *before screening*:

Duplicate records removed

(n =131)

Records marked as ineligible by automation tools (n = 0)

Records removed for other reasons (n = 0)

Records identified from*

Databases (n =266)

1. Scopus (n=84)
2. PubMed (n=69)
3. Web of science (n=113)

**Identification**

Records screened

(n =135)

Records excluded**

(n =113)

Reports sought for retrieval

(n =22)

Reports not retrieved

(n = 1)

**Screening**

Reports assessed for eligibility

(n =21)

Reports excluded:7

No/Wrong outcome (n = 3)

Conference Abstract (n = 2)

Wrong gene (n = 1)

Wrong location of cancer (n=1)

Retracted article (n=1)

Reports of studies included for Meta-analysis

(n =12)

**Included**

**Figure SF6: PRISMA diagram ERCC1 gene**
